# Differential protein analysis of saline-alkali promoting the oil accumulation in *Nitzschia palea*

**DOI:** 10.1186/s13068-023-02451-8

**Published:** 2024-01-28

**Authors:** Xintong Wang, Xianghong Meng, Yanlong Dong, Chunhua Song, Fengyang Sui, Xinxin Lu, Xiaoxue Mei, Yawen Fan, Yan Liu

**Affiliations:** 1 College of Life Sciences and Technology, Harbin Normal University, Harbin, China; 2 Key Laboratory of Biodiversity of Aquatic Organisms, Harbin Normal University, Harbin, China

**Keywords:** Saline-alkali, *Nitzschia palea*, Proteomics, Algae oil, Fatty acids, Freshwater diatoms

## Abstract

**Background:**

The increasingly severe salinization of the aquatic environment has led to serious damage to the habitats of aquatic organisms. Benthic diatoms are commonly employed as indicator species for assessing water quality and serve as a reflection of the overall health of the aquatic ecosystem. *Nitzschia palea* is a common diatom found in freshwater, with high oil content, rapid reproductive rate, and it is a commonly dominant species in various rivers.

**Results:**

The results showed that after 4 days (d) of saline-alkali stress, the cell density and chlorophyll a content of *Nitzschia palea* reached their maximum values. Therefore, we selected *Nitzschia palea* under 4 d stress for Tandem Mass Tag (TMT) quantitative proteomic analysis to explore the molecular adaptation mechanism of freshwater diatoms under saline-alkali stress. Totally, 854 proteins were enriched, of which 439 differentially expressed proteins were identified. Gene ontology (GO), Kyoto Encyclopedia of Genes and Genomes (KEGG), and subcellular fractionation analysis revealed that these proteins were mainly enriched in the photosynthesis pathway, citric acid cycle (TCA cycle), fatty acid synthesis, and glutathione cycle.

**Conclusions:**

This study aims to reveal the physiological, biochemical and proteomic mechanisms of salt and alkali tolerance and molecular adaptation of *Nitzschia palea* under different saline-alkali concentrations. This study showed that *Nitzschia palea* is one candidate of the environmental friendly, renewable bioenergy microalgae. Meantime, *Nitzschia* *palea* reveals for the proteome of the freshwater and provides the basis, it became a model algal species for freshwater diatoms.

**Supplementary Information:**

The online version contains supplementary material available at 10.1186/s13068-023-02451-8.

## Background

The salinization of the aquatic environment is becoming a widespread global environmental problem [1]. Moreover, it can precipitate biodiversity loss and disruption of food chains, consequently exerting detrimental effects on the structure and functionality of aquatic ecosystems, with further implications for human safety [2]. Research has demonstrated that a moderate level of salinity and alkalinity can be conducive to the growth of aquatic organisms, owing to their inherent osmoregulatory capacity. However, when the concentration of saline and alkali surpasses an organism's osmoregulatory threshold, its development and survival, become gravely imperiled [3, 4]. The microalgae are often an important component of aquatic ecosystems and also serve as food for plankton. Therefore, as the water environment changes, the growth of microalgae will also be affected accordingly [5].

Recently, the molecular mechanism of plant response to saline-alkali stress has become a research hotspot [6]. The salinization of the water impacts the structure and function of the membrane of microalgae cells, resulting in osmotic stress in various membrane systems of microalgae, thereby affecting the efficiency of photosynthesis utilization [7]. Studies have shown that *Microcystis aeruginosa* can tolerate saline-alkali stress to a certain extent by increasing the content of photosynthetic pigments [8]. Saline-alkali stress can induce the production of reactive oxygen species (ROS), such as superoxide anions, hydrogen peroxide, and hydroxyl radicals in microalgae cells [9]. Microalgae can scavenge ROS through enzymatic system and non-enzymatic system. Studies have shown that Aphanothece halophytica alleviated oxidative damage related to salt stress by increasing the activity of the superoxide dismutase (SOD), peroxidase (POD) and catalase (CAT) enzymes [10]. At the same time, microalgae will synthesize large numbers of fatty acids and carotenoids under saline-alkali stress [11]. The study showed that NaCl could increase the lipid content of *Dunaliella salina*; the ratio of oleic acid and palmitic acid of *Botryococcus braunii* increased as salinity increased [12, 13]. It was found that Na_2_CO_3_ could significantly promote the growth and oil accumulation of *Ankistrodesmus* sp [14]. Therefore, the successful development of salt-tolerant *Arthrospira platensis* not only solves the problems in open expansion culture but also uses saline-alkali water resources, which plays an important role in saving fresh water resources [15].

Importantly, proteomics studies provide new insights into the adaptive mechanisms of algae in response to different stresses. Studies have shown that 31 membrane-associated proteins of *Thalassiosira pseudonana* may be involved in the formation of diatom structures under silicate stress, and this is the first time that diatoms have been analysed using proteomics [16]. *Phaeodactylum tricornutum* as a model organism for diatom, the proteomics of is mainly focused on carbon and nitrogen metabolism, fatty acid and lipid synthesis of diatoms under N and P limiting conditions. The intracellular changes in *Phaeodactylum tricornutum* under different conditions were revealed at the protein level, such as the increase of plastidial palmitoyl- ACP desaturase, which is an important marker of carbon metabolism towards fatty acid and triacylglycerol synthesis [17–20]. *Dunaliella salina* is often used as a model algal species for halophilic algae and the molecular mechanisms of its salt tolerance indicated that salinity-regulated proteins were mainly involved in the Calvin cycle, energy production, protein synthesis/degradation, membrane structure stabilisation and signal transduction pathways [21, 22]. In *Fragilariopsis cyclindrus*, the synthesis of osmotic regulators and antioxidant enzymes is an important factor in the salinity domestication [23]. The studies of saline-alkali tolerance mechanism were focus on marine diatom mostly, researches in freshwater diatoms are wanting.

*Nitzschia palea* is a freshwater diatom that grows in various water bodies, such as lakes, reservoirs, and streams, and characteristic by its fast reproduction and high oil content. Research has shown that *Nitzschia palea* is a type of widely salt tolerant diatom, but the mechanism of its response to saline-alkali has not been fully investigated [24, 25]. In this study, *Nitzschia palea* were used as the research object to compare and analyze their growth and physiological changes under different saline-alkali stress concentrations, as well as their changes in oil content. Through differential expression proteomics analysis, we aim to reveal the molecular response mechanism of *Nitzschia palea* to saline-alkali stress, and provide data support for further research on the saline-alkali tolerance mechanism of diatoms.

## Materials and methods

### Algal strain and culture conditions

*Nitzschia palea* was collected and purified from the Key Laboratory of Aquatic Biology in Heilongjiang Province, Harbin Normal University, and cultured in WC medium (Table S1) [26].

#### Experimental design

The mixed components of saline-alkali are mainly NaCl and NaHCO_3_. The average ratio of NaCl to NaHCO_3_ is 1:2. The concentration settings are as follows, as shown in Table 1:

**Table 1:** Experimental concentration setting

Group	NaCl (mmol·L^−1^)	NaHCO_3_ (mmol·L^−1^)
Control Check (CK)	0	0
30 mM	10	20
60 mM	20	40
90 mM	30	60

Note: Control Check (CK: 0 mmol·L^−1^ NaCl and 0 mmol·L^−1^ NaHCO_3_) In this study, *Nitzschia palea* at the logarithmic growth phase (approximately 4 days) were selected for the experiment. The cultivation conditions are temperature 25 °C, 12-h light–dark cycle, and light intensity 6000 lx. Shake the algal solution 3 to 6 times per day to prevent cell aggregation and promote algae growth.

### Algal growth and physiological parameters

Two mL of microalgae suspension were taken from each treatment group, measure the transmittance at a wavelength of 680 nm, calculate the cell count based on the standard curve, repeat the procedure three times for each treatment group, and record the changes in the cell count of *Nitzschia palea* over a period of 6 days.

Mix 10 mL of algal cells with 5 mL of anhydrous ethanol solution. The sample was extracted in darkness at 4 ℃ for 24 h, and then centrifuged (at 3500 rpm, 10 min, 4 ℃) again to collect the supernatant. Record the OD_646_ and OD_663_ values to calculate the chlorophyll a ratio using the following formula:

$${\text{Concentration}}\,{\text{of}}\,{\text{chlorophyll}}\,{\text{a}}\,\left( {{\text{mg}} \cdot {\text{L}}^{{ - {1}}} } \right)\, = \,{12}.{21} \cdot {\text{OD}}_{{{663}}} \,{-}\,{2}.{81} \cdot {\text{OD}}_{{{646}}}$$

For other physiological parameters, we mainly refer to the "Principles and Techniques of Plant Physiology and Biochemistry Experiments".

### Determination of oil content and fat composition

The method for oil extraction involves drying the algae powder through acid hydrolysis and then extracting it using the chloroform–methanol extraction method [27].

The fatty acid composition was analyzed using Agilent gas chromatography-mass spectrometer. Chromatographic column: TG-5MS column (30.0 m × 0.25 mm × 0.25 μm); Carrier gas: He (purity ≥ 99.999%); injection volume 1 μL; injection port temperature 290 ℃; carrier gas: He; the flow rate was 1.2 ml·min^−1^; temperature rise program: maintain 80 ℃ for 1 min, increase the temperature to 200 ℃ at a rate of 10 ℃·min^−1^, maintain a rate of 5 ℃·min^−1^ until reaching 250 ℃ and finally increase the temperature to 270 ℃ at a rate of 2 ℃·min^−1^ and maintain for 3 min.

Ion source: EI; Ion source temperature: 280 ℃. Ion energy mode: Use tuning settings; Ion energy (EV): 70. Detector setting: Use gain factor; Solvent delay: 5 min; Quality scanning range: 30 ~ 400.

### Proteomic analysis

#### Protein extraction and digestion

The protein extraction and digestion process involved the use of SDT (4% SDS, 100 mM Tris–HCl, 1 mM DTT, pH 7.6) buffer for sample lysis and protein extraction. The concentration of protein was determined using the BCA Protein Assay Kit (Bio-Rad, USA). Protein digestion was carried out utilizing trypsin following the filter-aided sample preparation (FASP) procedure outlined by Matthias Mann [28]. The peptides resulting from the digestion of each sample were subsequently purified using C18 Cartridges (Empore^™^ SPE Cartridges C18 (standard density), bed I.D. 7 mm, volume 3 ml, Sigma, Germany), concentrated through vacuum centrifugation, and reconstituted in 40 µl of 0.1% (v/v) formic acid [29, 30]

#### SDS-PAGE

20 µg of protein for each sample were mixed with 5X loading buffer, respectively, and boiled for 5 min. The proteins were separated on 12.5% SDS-PAGE gel (constant current 14 mA, 90 min). Protein bands were visualized by Coomassie Blue R-250 staining [31].

#### Labeling

100 μg peptide mixture of each sample was labeled using TMT reagent according to the manufacturer’s instructions (Thermo Scientific, America).

#### Protein identification using nano-HPLC–MS/MS

LC–MS/MS analysis was performed on a Q Exactive mass spectrometer (Thermo Scientific, Q Exactive HF-X) that was coupled to Easy nLC (Proxeon Biosystems, now Thermo Fisher Scientific) for 60/90 min. The peptides were loaded onto a reverse phase trap column (Thermo Scientific Acclaim PepMap100, 100 μm 2 cm, nanoViper C18) connected to the C18-reversed phase analytical column (Thermo Scientific Easy Column, 10 cm long, 75 μm inner diameter, 3 μm resin) in buffer A (0.1% Formic acid) and separated with a linear gradient of buffer B (84% acetonitrile and 0.1% formic acid) at a flow rate of 300 nl/min controlled by Intelli Flow technology. The mass spectrometer was operated in positive ion mode. MS data were acquired using a data-dependent top10 method dynamically choosing the most abundant precursor ions from the survey scan (300–1800 m z^−1^) for HCD fragmentation. Automatic gain control (AGC) target was set to 3e6, and maximum inject time to 10 ms. Dynamic exclusion duration was 40.0 s. Survey scans were acquired at a resolution of 70,000 at m z^−1^ 200 and resolution for HCD spectra was set to 17,500 at m z^−1^ 200, and isolation width was m z^−1^. Normalized collision energy was 30 eV and the underfill ratio, which specifies the minimum percentage of the target value likely to be reached at maximum fill time, was defined as 0.1%. The instrument was run with peptide recognition mode enabled.

#### Identification and quantitation of proteins

The MS raw data for each sample were searched using the MASCOT engine (Matrix Science, London, UK; version 2.2) embedded into Proteome Discoverer 1.4 software for identification and quantitation analysis.

### Real-time quantitative PCR (qRT-PCR)

Primer Premier 5.0 software was used to design qRT-PCR-specific primers for *nppbsc, npsos1, npgapdh, npfabg, npsdha * and *nplscl* (Table S2). The *npactin* was used as the internal reference gene, and qRT-PCR was performed on *nppbsc, nppbsl, npsos1, npsos2, npsos3* and *npfabg*. Three biological replicates and three technical replicates were carried out for each sample, and the relative expression of the genes was calculated by adopting the 2^−ΔΔCt^ method [32].

### Data analysis

All data were statistically analyzed using GraphPad Pirsm software, and the significance of differences between groups was analyzed by one-way ANOVA, with results considered to be highly significant at *P* < 0.01 and significant at *P* < 0.05.

## Results

### Effect of saline-alkali stress on the growth of *Nitzschia palea*

**Fig. 1:**
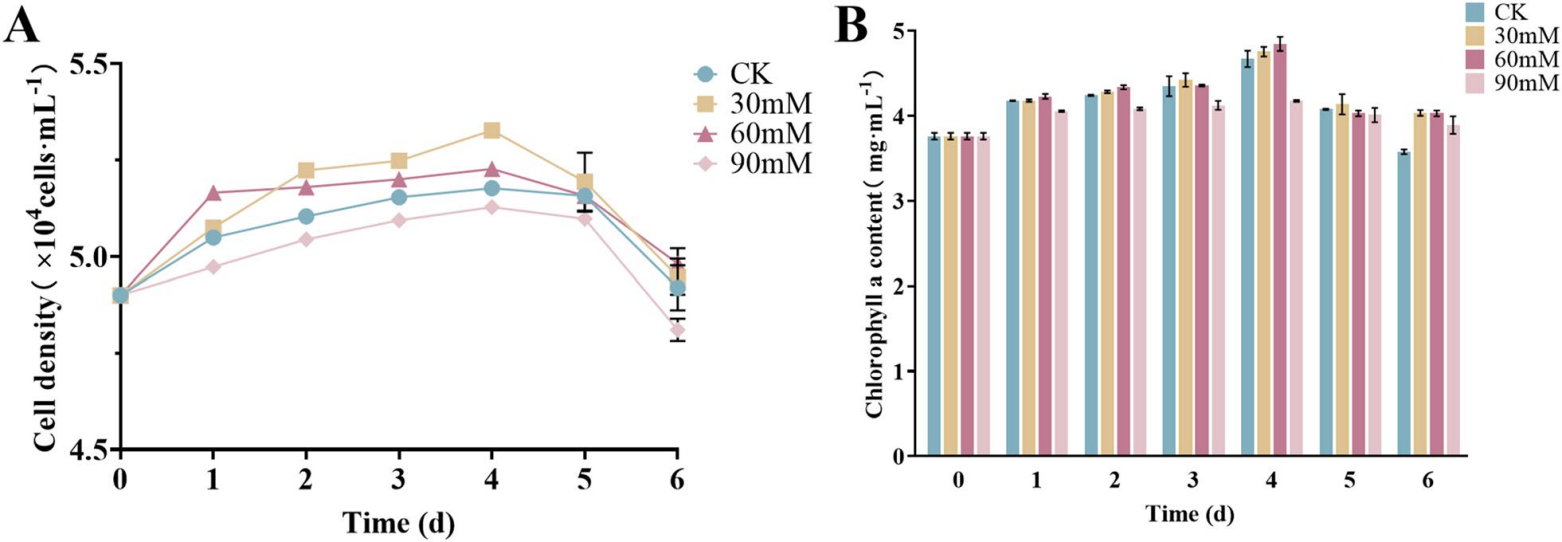
Effect of saline-alkali stress on the growth of *Nitzschia palea*. **A** Cell density of *Nitzschia palea*; **B** chlorophyll a content of *Nitzschia palea*. The data are presented in the form of mean ± SE (*n* = 3)

Cell density of microalgae is an important indicator of microalgal response to stress [33]. The experimental results showed that cell density exhibited an increasing and then decreasing trend with the increase in saline-alkali concentration. On day 4, the cell density of microalgae peaked at 30 mM treatment (Fig. 1A).

Chlorophyll a is present in all microalgae and serves as a reflection of their growth [34]. The experimental results indicated that the chlorophyll a content exhibited an increasing and then decreasing trend, mirroring the trend observed in cell density (Fig. 1B). On day 4, the chlorophyll a content peaked at the 60 mM treatment. Therefore, *Nitzschia palea* treated with various saline-alkali concentrations for 4 days will be selected for subsequent experiments.

### Effect of saline-alkali stress on oil content and fatty acid composition of *Nitzschia palea*

**Fig. 2:**
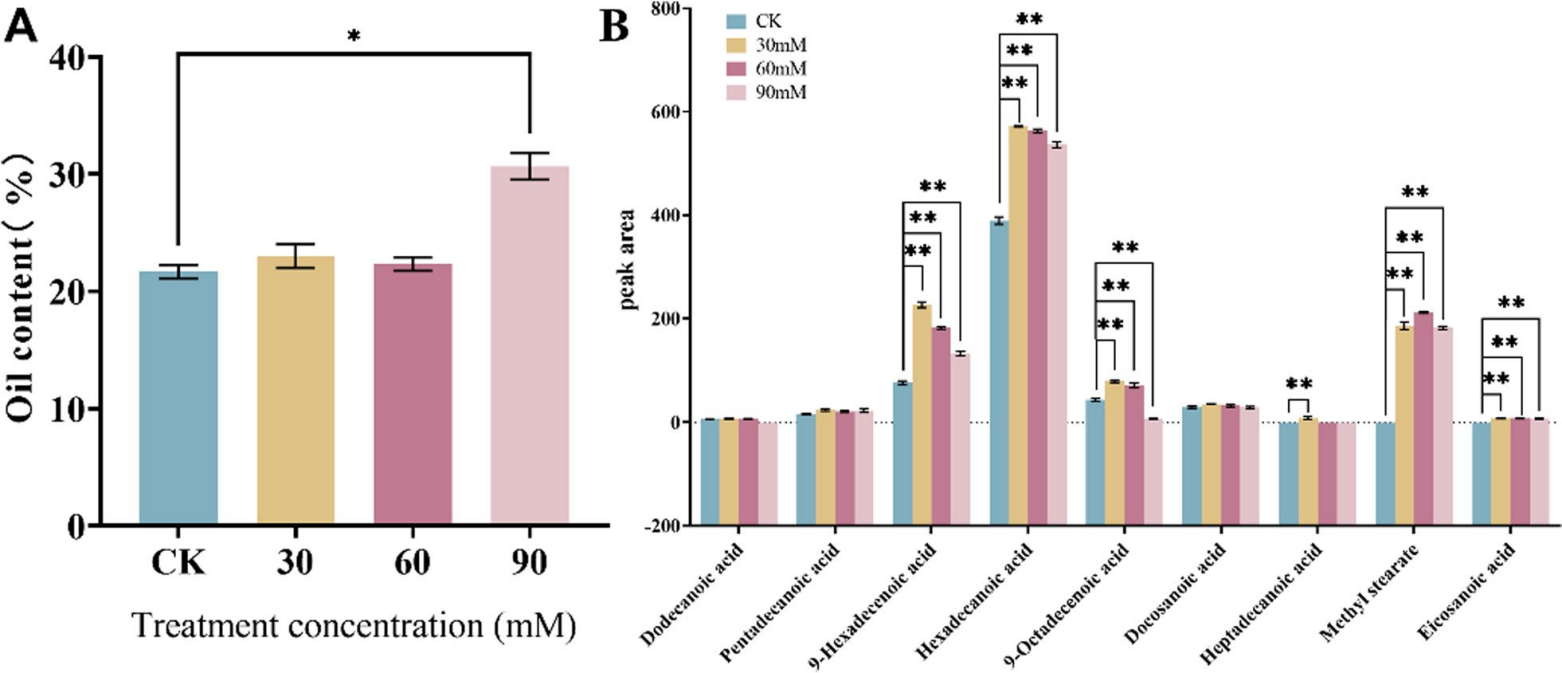
Effect of saline-alkali stress on oil content and fatty acid composition of *Nitzschia palea*. **A** Oil content of *Nitzschia palea*; **B** peak area of *Nitzschia palea*. The data are presented in the form of mean ± SE (*n* = 3). * and ** indicate values that differ significantly from controls at *P* < 0.05 and *P* < 0.01, respectively, according to one-way ANOVA

Oils are the main energy storage products of microalgae, which can reflect the physiological activity and health of microalgae under the stress of different saline-alkali concentrations [11]. As shown in Fig. 2A, the change in oil content of *Nitzschia palea* increased with the increase in saline-alkali concentration after 4 days of saline-alkali stress treatment. At the 90 mM treatment, the oil content increased significantly (*P* < 0.05) compared with the control group (CK).

Microalgae under saline-alkali stress produce different physiological mechanisms, such as intracellular and extracellular uptake of sodium ions, which in turn have an effect on the fluidity of their cell membranes [12]. This effect can result in changes in fatty acid composition. The results of the fatty acid composition analysis in this study are shown in (Additional file 1: Figure S1 and Table S3). Hexadecanoic acid (C16:0) accounts for a relatively high percentage of the total fatty acid content. 9-hexadecenoic acid (C16:1) showed an increasing and then decreasing trend, with the peak area being highest at the 30 mM treatment. Subsequent to this, there was an accumulation of methyl stearate (C18:0) in microalgal cells under saline-alkali stress, as shown in Fig. 2B.

### Effects of saline-alkali stress induced physiological changes in *Nitzschia palea*

**Fig. 3:**
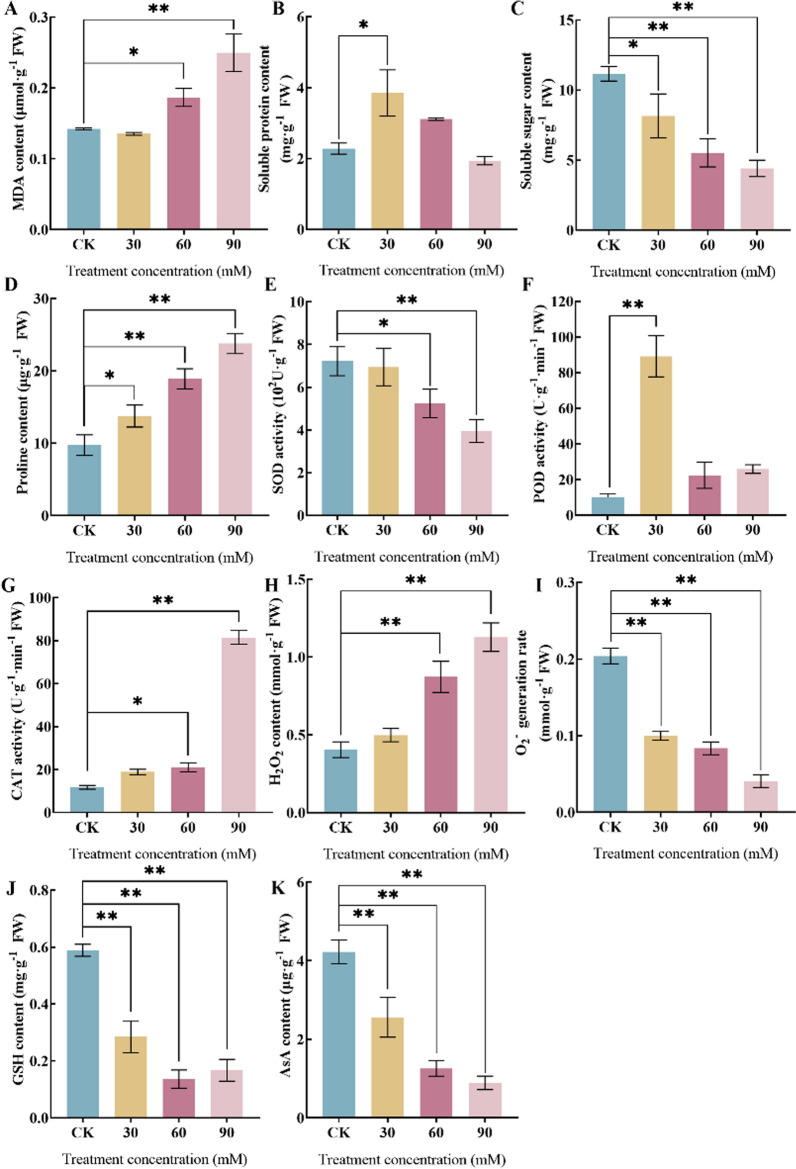
Physiological changes of MDA content (**A**), Soluble protein content (**B**), Soluble sugar content (**C**), SOD activities (**D**), POD activities (**E**), CAT activities (**F**), Proline content (**G**), H_2_O_2_ content (**H**), O_2_^−^ generation rate (**I**), GSH contents (**J**) and AsA contents (**K**) *Nitzschia palea* under saline-alkali stress. The data are presented in the form of mean ± SE (*n* = 3). * and ** indicate values that differ significantly from controls at *P* < 0.05 and *P* < 0.01, respectively, according to one-way ANOVA

Microalgae under saline-alkali stresses appear to have an imbalance of intracellular osmotic pressure and changes in membrane structure, leading to damage to the cell membrane. As MDA is the end product of lipid peroxidation, its level characterises the degree of cell membrane damage and the strength of cellular resistance [35]. The MDA content gradually increased with increasing saline-alkali concentration (Fig. 3A). The content of MDA showed a significant increase (*P* < 0.05) under the 60 mM treatment compared with CK.

When subjected to saline-alkali stress, microalgae rapidly produce and accumulate osmoregulatory substances, such as soluble proteins, soluble sugars, and proline to balance the intracellular osmotic pressure and resist the damage caused by various external stresses [36]. The increase in soluble protein content facilitates the immobilization of water molecules, thereby regulating the intracellular and extracellular osmotic pressure and maintaining the integrity of the cell membrane [37]. The soluble protein content gradually increases with increasing saline-alkali concentration (Fig. 3B). Under the 30 mM treatment, the soluble protein content significantly increases (*P* < 0.05) compared with CK. On the other hand, the soluble sugar content decreases gradually with increasing saline-alkali concentration (Fig. 3C). The soluble sugar content is significantly decreased (*P* < 0.05) under the 30 mM treatment compared with CK. Moreover, under the 60 mM and 90 mM treatments, the soluble sugar content is significantly decreased (*P* < 0.01) compared with CK.

Proline accumulation is an effective way for plant bodies to resist osmotic stress and maintain the normal physiological functions of cells by regulating their osmotic potential and stabilizing their protein structure [38]. Proline content gradually increases with increasing saline-alkali concentration (Fig. 3D). Under the 30 mM treatment, proline content significantly increases (*P* < 0.05) compared with CK. Moreover, under the 60 mM and 90 mM treatments, proline content is significantly increased (*P* < 0.01) compared with CK.

Microalgae under saline-alkali stress treatment causes ROS accumulation in the short term, generating lipid peroxidation products. Simultaneously, it stimulates the algal antioxidant enzyme system to produce antioxidant enzymes (such as SOD, POD and CAT, etc.) to mitigate the cellular damage caused by ROS under saline-alkali stress [39–41]. SOD activity shows a decreasing trend with increasing saline-alkali concentration (Fig. 3E). Under the 60 mM treatment, SOD activity is significantly decreased (*P* < 0.05) compared with CK. Moreover, under the 90 mM treatment, SOD activity is significantly decreased (*P* < 0.01) compared with CK.

Peroxidase (POD) is an effective quencher of ROS, and the level of its activity measures the strength of the cell's resistance to oxidative damage [42]. POD activity showed an increasing trend with increasing saline-alkali concentration (Fig. 3F). POD activity was significantly increased (*P* < 0.01) under 30 mM treatment compared with CK.

Catalase (CAT) activity showed an increasing trend with increasing saline-alkali concentration (Fig. 3G). CAT activity increased significantly (*P* < 0.05) under 60 mM treatment compared with CK. CAT activity was significant (*P* < 0.01) under 90 mM treatment compared with CK.

SOD can catalyze the dismutation of superoxide in microalgae cells to produce O_2_^−^ and H_2_O_2_ [43]. The content of H_2_O_2_ gradually increased with increasing saline concentration (Fig. 3H). H_2_O_2_ content was significant (*P* < 0.01) increased under 60 mM and 90 mM treatments compared with CK. The superoxide anion rate gradually decreased with increasing saline-alkali concentration (Fig. 3I). The rate of superoxide anion was significantly decreased (*P* < 0.01) under 30 mM, 60 mM and 90 mM treatments compared with CK.

Glutathione (GSH) will be involved in the absorption and transport of amino acids and maintain the integrity of cell membranes and scavenge-free radicals in the body [44]. GSH content gradually decreased with increasing saline-alkali concentration (Fig. 3J). GSH content was significantly decreased (*P* < 0.01) under 30 mM, 60 mM and 90 mM treatments compared with CK.

Ascorbic acid (AsA) has very important biological functions in plant growth and development processes, such as antioxidant scavenging of free radicals, photosynthesis, cell growth and division [45]. AsA content gradually decreased with increasing saline-alkali concentration (Fig. 3K). AsA content decreased significantly (*P* < 0.01) under 30 mM, 60 mM and 90 mM treatments compared with CK.

### Extraction and assay of protein from *Nitzschia palea*

To analyse the differences in the protein expression in *Nitzschia palea* under different saline-alkali concentrations, samples were taken at 4 d of saline-alkali stress treatment for quantitative analysis using TMT. SDS-PAGE electrophoresis was used to separate the proteins under different saline-alkali concentrations. The electrophoresis results showed clear protein bands without any degradation in both the saline-alkali stress and control groups, and the protein quantity met the requirements for subsequent experiments. These results indicate that formal experiments can be conducted in the future (Additional file 1: Figure S2). The protein content of *Nitzschia palea* used for proteomics analyses met the requirements of the TMT test (Additional file 1: Table S4).

### Proteomic analysis of *Nitzschia palea* under saline and saline-alkali stress

A total of 854 proteins were identified in this study (Additional file 1: Figure S3D). The largest number of peptides after enzymatic digestion were those containing 7–13 amino acid residues (Additional file 1: Figure S3A). The coverage of the peptide sequences shows that proteins in the 0–5% and 5–10% coverage ranges have the largest ratio of 0 cases, while the proportion of proteins in the 40–45% range is the smallest (Additional file 1: Figure S3B). As shown in Additional file 1: Figure S3C, the molecular weights of all the proteins identified were mostly concentrated in the range of 10–90 kDa.

**Fig. 4:**
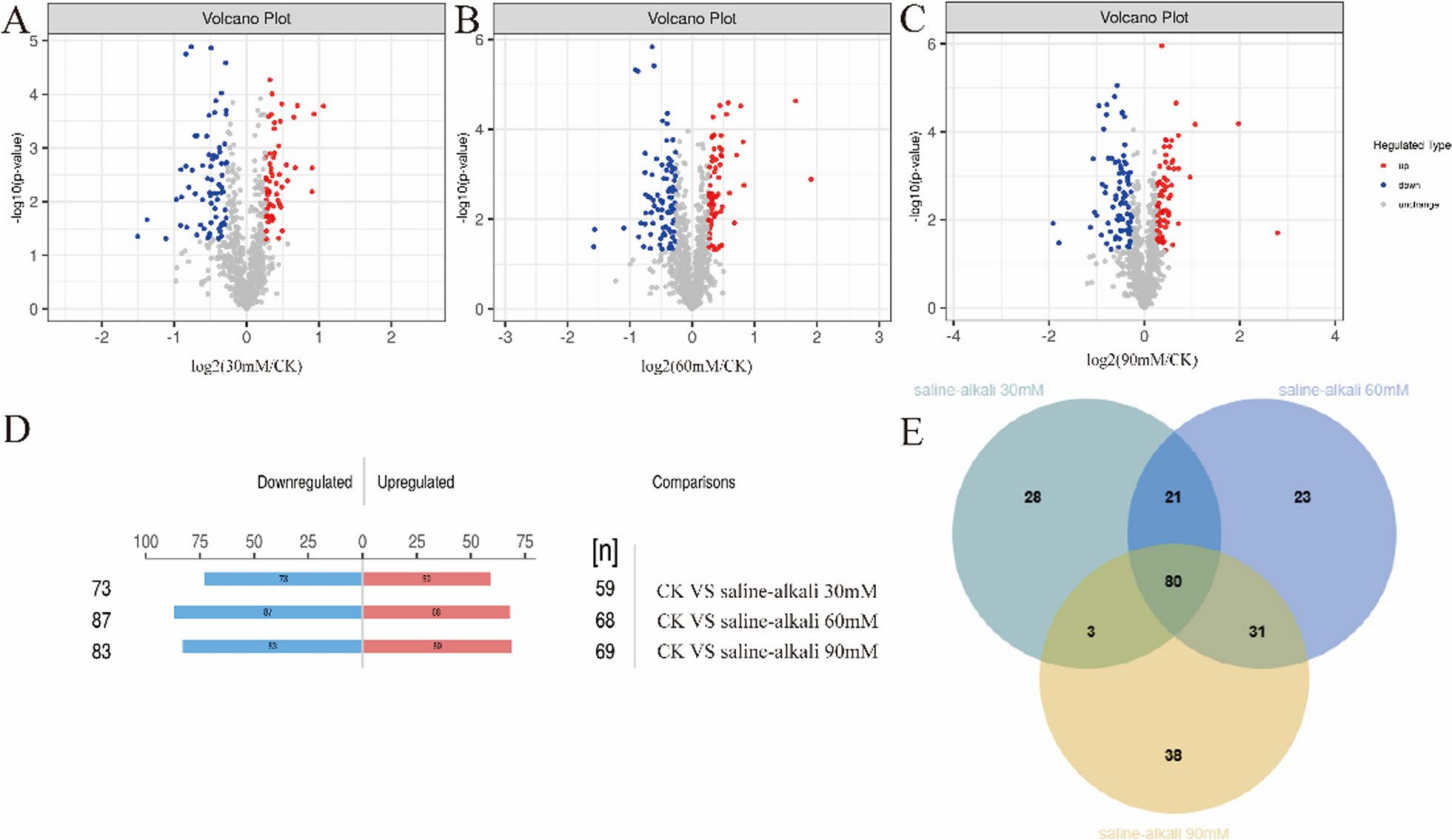
Proteomic analysis of *Nitzschia palea*. **A**–**D** Diagrams showing for each quantified protein the change in abundance between treatment (saline-alkali) and the control (CK). Most proteins (grey points) did not change in abundance between the two culture conditions. Significantly regulated proteins are depicted as red/blue points, and red/blue points indicate significantly increased/decreased proteins; **E** Venn diagram with the number of proteins identified in *Nitzschia palea* for the group of untreated CK, 30 mM, 60 mM, 90 mM

We selected the proteins with a *P* < 0.05 and ratio ≥ 1.2 as differential proteins. The distribution of differential proteins in two-by-two comparisons of *Nitzschia palea* with saline-alkali stress for 4 d at 30 mM, 60 mM, and 90 mM, compared with CK, is shown in Fig. 4. It demonstrates the differences in the number of differential proteins in different samples. After screening, a total of 439 differential proteins were detected in *Nitzschia palea* treated for 4 d with different concentrations of saline-alkali stress. There were a total of 132 differential proteins between CK and treatment 30 mM, including 59 up-regulated proteins and 73 down-regulated proteins; 155 differential proteins between CK and treatment 60 mM, including 68 up-regulated proteins and 87 down-regulated proteins; and 152 differential proteins between CK and treatment 90 mM, including 69 up-regulated proteins and 83 down-regulated proteins (Fig. 4D)."A Wayne diagram analysis of the differential proteins showed that there were a total of 80 differential proteins between the different concentrations of saline treatments of *Nitzschia palea*. (Fig. 4E).

### Functional annotation of *Nitzschia palea* proteins in different saline-alkali treatments

#### GO enrichment analysis of differentially expressed proteins (DEPs) under saline-alkali treatments

**Fig. 5:**
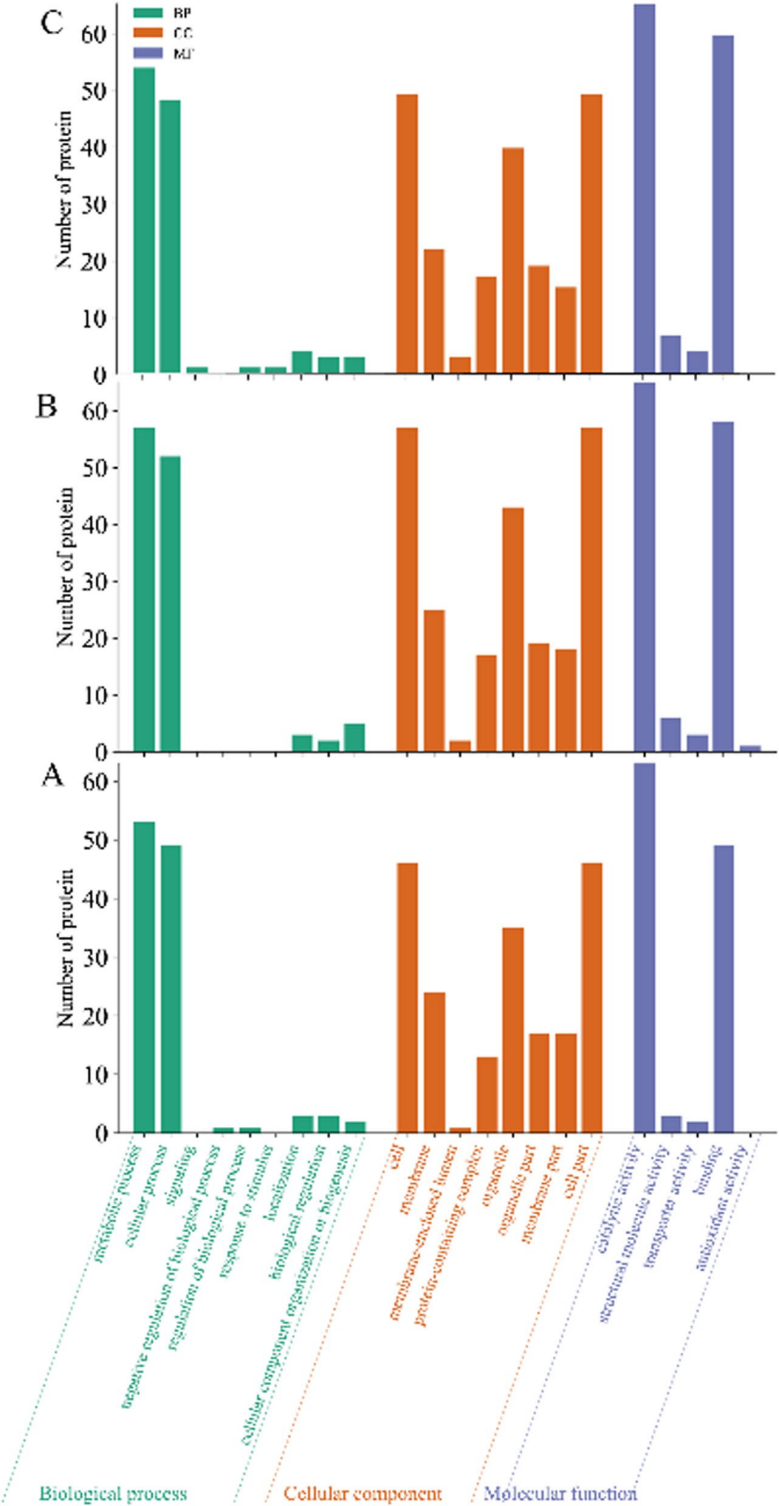
GO enrichment analysis of DEPs. **A** GO functional classification of differential proteins between CK and 30 mM; **B** GO functional classification of differential proteins between CK and 60 mM; **C** GO functional classification of differential proteins between CK and 90 mM

As shown in Fig. 5, the functional enrichment analysis of differential protein GOs for different concentrations of saline-alkali treatment of *Nitzschia palea* revealed that these proteins corresponded to biological processes, cellular components, and molecular functions.

In the treatment of 30 mM compared with CK, mainly involved in the metabolic process (GO:0008152, 53) and in the cellular process (GO:0009987, 49) in the biological process. The cellular components were mainly cell (GO:0005623, 46), cell part (GO:0044464, 46), organelle (GO:0043226, 35), and membrane (GO:0016020, 24). The molecular functions of these proteins were mainly catalytic activity (GO:0003824, 63) and binding (GO:0005488, 49).

In the treatment of 60 mM compared with CK, mainly involved in the metabolic process (GO:0008152, 57) and in the cellular process (GO:0009987, 52) in the biological process. The cellular components were mainly cell (GO:0005623, 57), cell part (GO:0044464, 57), organelle (GO:0043226, 43), and membrane (GO:0016020, 25). The molecular functions of these proteins were mainly catalytic activity (GO:0003824, 65) and binding (GO:0005488, 58).

In the treatment of 90 mM compared with CK, mainly involved in the metabolic process (GO:0008152, 57) and in the cellular process (GO:0009987, 51) in the biological process. The cellular components were mainly cell (GO:0005623, 52), cell part (GO:0044464, 52), organelle (GO:0043226, 42), and membrane (GO:0016020, 23). The molecular functions of these proteins were mainly catalytic activity (GO:0003824, 69) and binding (GO:0005488, 63).

### KEGG enrichment analysis of DEPs under saline-alkali treatments

**Fig. 6:**
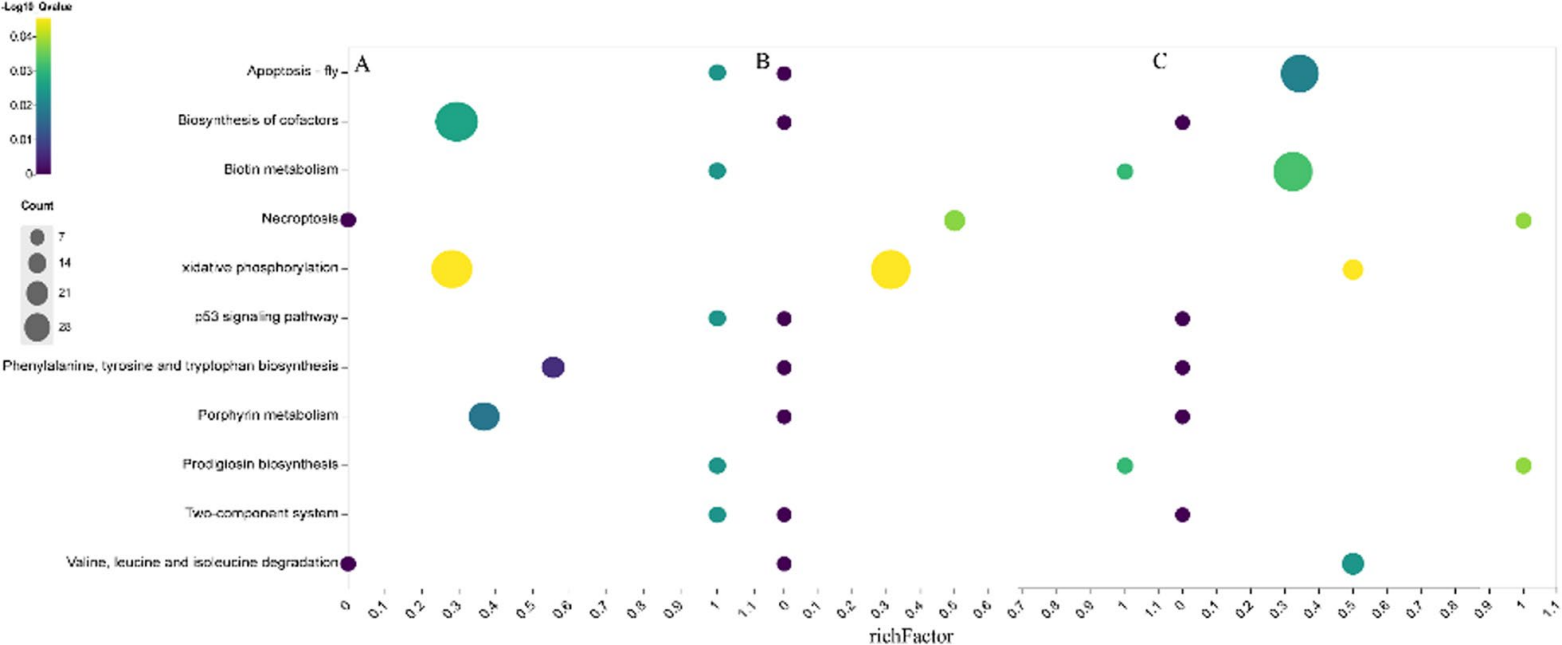
Pathway of differential protein enrichment between different statistical analysis of *Nitzschia palea*. **A** Significantly enriched pathways in 30 mM treatment; **B** significantly enriched pathways in 60 mM treatment; **C** significantly enriched pathways in 90 mM treatment

KEGG annotation analysis of proteins from *Nitzschia palea* under 30 mM treatment showed that these proteins were enriched for a total of 63 pathways. Nine pathways were significantly enriched (*P* < 0.05, Fig. 6A), mostly Phenylalanine, Tyrosine and tryptophan biosynthesis (ko00400), Porphyrin metabolism (ko00860), Biosynthesis of cofactors (ko01240), Oxidative phosphorylation (ko00190).

KEGG annotation analysis of proteins from *Nitzschia palea* under 60 mM treatment showed that these proteins were enriched for a total of 70 pathways. Four pathways were significantly enriched (*P* < 0.05, Fig. 6B) for Biotin metabolism (ko00780), Necroptosis (ko04217), Oxidative phosphorylation (ko00190), Prodigiosin biosynthesis (ko00333).

KEGG annotation analysis of proteins from *Nitzschia palea* under 90 mM treatment showed that these proteins were enriched for a total of 75 pathways. Six pathways were significantly enriched (*P* < 0.05, Fig. 6C) for Apoptosis-fly (ko04214), Biotin metabolism (ko00780), Oxidative phosphorylation (ko00190), Valine, leucine, and Isoleucine degradation (ko00280).

### Analysis of functional structural domains of proteins of *Nitzschia palea* under saline-alkali treatments

Functional structural domain annotation analysis in *Nitzschia palea* under 30 mM treatment showed that 854 proteins were enriched in a total of 155 functional structural domains. Among them, six were significantly enriched (*P* < 0.05, Table 2), including Glyceraldehyde 3-phosphate dehydrogenase, C-terminal (PF02800), glyceraldehyde 3-phosphate dehydrogenase, NAD-binding domain (PF00044), and Pyridoxal-phosphate dependent enzyme (PF00291).

**Table 2:** Statistics on the number of protein functional domains

Domain_id	Domain_Name	CK VS 30 mM		CK VS 60 mM		CK VS 90 mM	
		P value	count	P value	count	P value	count
PF02310	B12-binding domain	0	0	0	0	0.03	2
PF02492	CobW/HypB/UreG, nucleotide-binding domain	0	0	0	0	0.03	2
PF16211	C-terminus of histone H2A	0.02	2	0.03	2	0	0
PF13561	Enoyl-(Acyl carrier protein) reductase	0.02	2	0.03	2	0.03	2
PF02800	Glyceraldehyde 3-phosphate dehydrogenase, C-terminal domain	0.03	5	0	0	0.04	5
PF00044	Glyceraldehyde 3-phosphate dehydrogenase, NAD-binding domain	0.03	5	0	0	0.04	5
PF01078	Magnesium chelatase, subunit ChlI	0	0	0.03	2	0	0
PF01849	NAC domain	0	0	0.03	2	0	0
PF03446	NAD-binding domain of 6-phosphogluconate dehydrogenase	0	0	0	0	0.03	2
PF00421	Photosystem II protein	0.02	2	0	0	0	0
PF00291	Pyridoxal-phosphate dependent enzyme	0.01	4	0	0	0	0
PF03947	Ribosomal Proteins L2, C-terminal domain	0	0	0.03	2	0.03	2
PF00181	Ribosomal Proteins L2, RNA-binding domain	0	0	0.03	2	0.03	2

Functional structural domain annotation analysis in *Nitzschia palea* under 60 mM treatment showed that 854 proteins were enriched in a total of 187 functional structural domains. Among them, 6 were significantly enriched (*P* < 0.05, Table 2), including C-terminus of histone H2A (PF16211), enoyl-(Acyl carrier protein) reductase (PF13561), ribosomal proteins L2, C-terminal domain (PF03947), ribosomal proteins L2, RNA-binding domain (PF00181).

Functional structural domain annotation analysis in *Nitzschia palea* under 90 mM treatment showed that 854 proteins were mainly enriched in a total of 192 functional structural domains. Among them, 8 were significantly enriched (*P* < 0.05, Table 2) as Glyceraldehyde 3-phosphate dehydrogenase, C-terminal (PF02800), Glyceraldehyde 3-phosphate dehydrogenase, NAD-binding domain (PF00044).

### Analysis of the subcellular localisation in *Nitzschia palea* under saline-alkali treatments

The proteins of *Nitzschia palea* under 30 mM treatment were mainly localized in chloroplast and Plastid sites. A few proteins were localized in Membrane, Multi-pass membrane protein and Mitochondrion inner membrane (Additional file 1: Figure S5).

The proteins of *Nitzschia palea* under 60 mM treatment were mainly localized in chloroplast and Plastid sites. A few proteins were localized in Membrane, Multi-pass membrane protein and Nucleus.

The proteins of *Nitzschia palea* under 90 mM treatment were mainly localized in chloroplast, Plastid, Membrane and Multi-pass membrane protein. A few are localized to Chloroplast thylakoid membrane, Mitochondrion inner membrane and Nucleus.

### Differential protein metabolic mapping

**Fig. 7:**
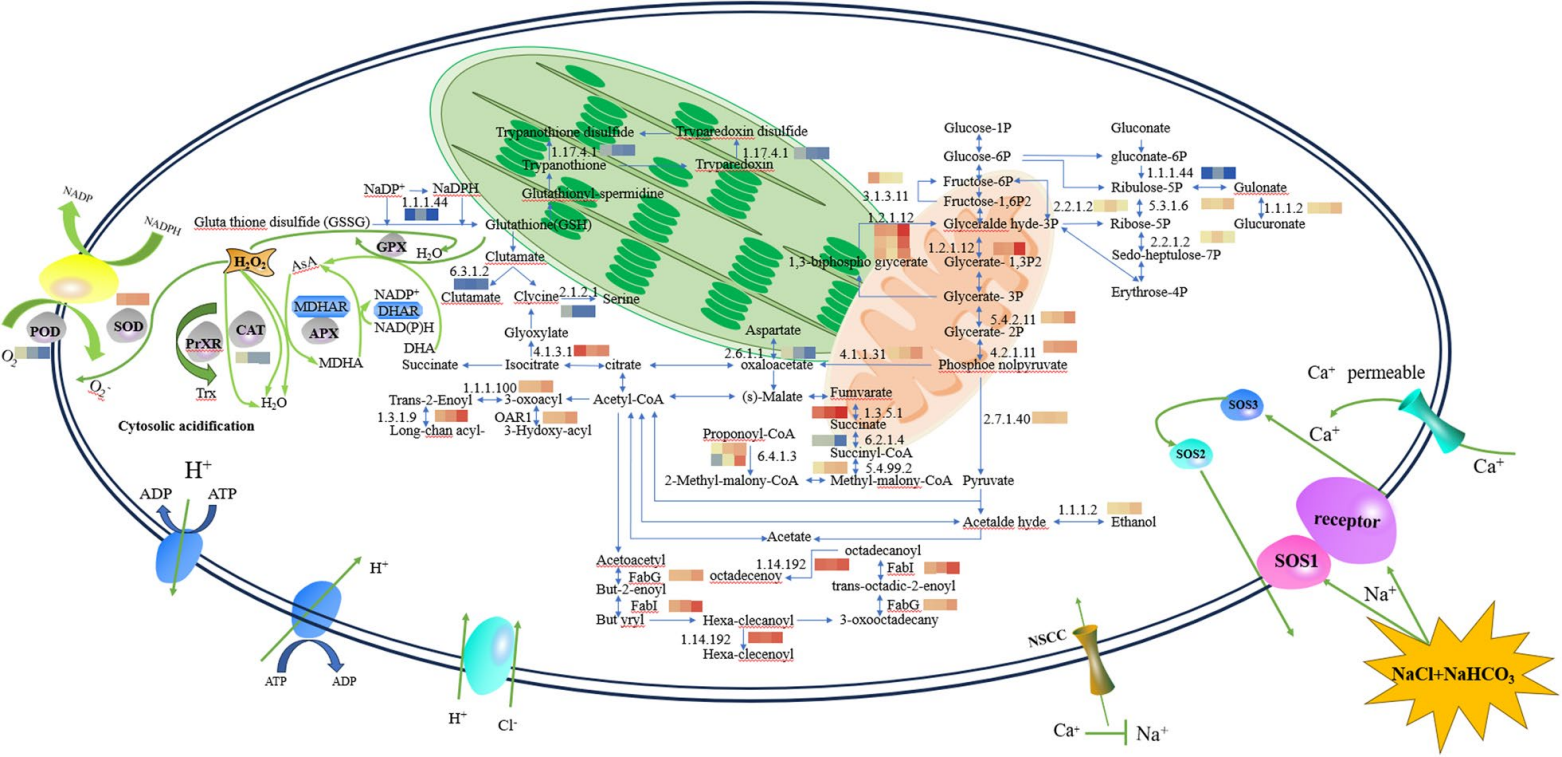
Main metabolic pathway network related to differentially expressed proteins (DEPs)

After GO and KEGG annotation, protein functional structural domain and subcellular localization analysis, differential proteins *nppbsc, npsos1, npsos2, npsos3, npcdk1, nppgd, npsucd, nplsc1, nppbs27, nppmca, npant, npgme, npfabg, npfabi, npsdha, npsdhb, npgapdh, nppgam* and *npeno* are primarily involved in the following biological processes: metabolism, stress and defence, photosynthesis, transmembrane transport, and ROS signalling. The largest category is metabolism, which primarily involves the glycolytic pathway, carbon fixation by photosynthetic organisms, the citric acid cycle (TCA cycle) and fatty acid biosynthesis. Figure 7 shows the regulatory mapping of metabolism-related proteins in the proteomic analysis of *Nitzschia palea* under saline-alkali stress n (Table S5). The metabolic mapping was obtained based on GO functional annotation and KEGG annotation of differentially expressed proteins, and combined with the relevant literature on the study of plant resistance to saline-alkali stress.

### qRT-PCR analysis of differentially expressed proteins in *Nitzschia palea*

**Fig. 8:**
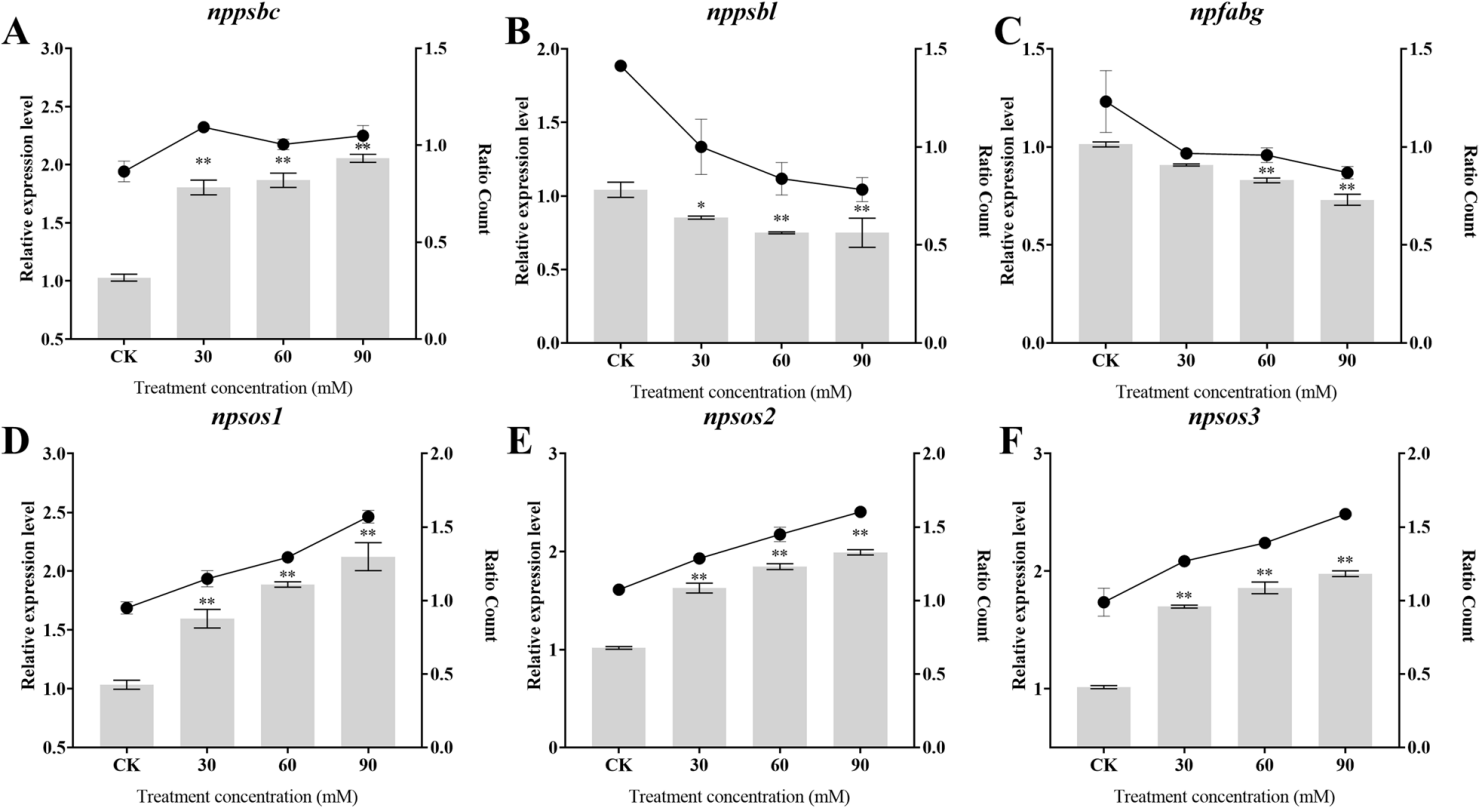
Effect of saline-alkali treatment on *nppsbc* (**A**), *nppsbl* (**B**), *npfabg* (**C**),*npsos1* (**D**), *npsos2* (**E**) and *npsos3* (**F**) expression of *Nitzschia palea*. The data are presented in the form of mean ± SE (*n* = 3). * and ** indicate values that differ significantly from controls at *P* < 0.05 and *P* < 0.01, respectively, according to one-way ANOVA

In this study, we further validated the accuracy and reliability of mRNA-seq data using qRT-PCR to measure the gene expression of *Nitzschia palea* treated with different saline concentrations, including *nppsbc, nppsbl, npsos1, npsos2, npsos3* and *npfabg*. These proteins are mainly enriched in the glycolytic pathway, carbon fixation by photosynthetic organisms, TCA cycle and fatty acid biosynthesis. The results showed that the expression of proteins *nppsbc, npsos1, npsos2* and *npsos3* was significantly up-regulated (*P* < 0.01), while the expression of protein *nppsbl* and *npfabg* was significantly down-regulated (*P* < 0.05) in the *Nitzschia palea* treated with saline-alkali stress compared with CK, which is in agreement with the changes observed in the mRNA-seq date (Fig. 8). These results indicate that mRNA-seq data are reliable.

## Discussion

### Effect of saline-alkali stress on the growth and chlorophyll a content of *Nitzschia palea*

Compared to single salt stress, saline-alkali stress not only causes ionic toxicity, osmotic stress and oxidative damage to plants, but also produces high pH stress, which affects the growth and development of algae and results in more complex and severe damage to algae. *Nitzschia palea* is a broadly saline diatom [24]. Studies have demonstrated that *Nitzschia* sp. MD1 exhibits strong tolerance to high alkali and salinity, and the biomass of microalgae significantly increases under high alkaline and salinity cultivation conditions [46]. In our study, the growth of *Nitzschia palea* treated with saline-alkali for 4 days were significantly increased, consistent with previous reports. This further confirms the tolerance of *Nitzschia palea* to saline-alkali conditions. Therefore, we selected different saline-alkali concentrations for a 4-day investigation into the protein-level response mechanism to saline-alkali in *Nitzschia palea*.

Chloroplasts are important organelles for energy conversion and photosynthesis in plants, and chlorophyll is an important photosynthetic pigment for photosynthesis in plants, that reflects plant resistance to some extent. The results of this research showed that a significant increase in the chlorophyll a content of *Nitzschia palea* treated with 60 mM was observed under saline-alkali treatment. In the proteomic analysis, six proteins were predominantly enriched in the photosynthesis pathway and *nppsb27* was apparently one of the proteins important for maintaining the efficient repair of PSII [47]. The expression of proteins *nppsb27* and *nppsbl* was significantly down-regulated, while the expression of *nppsbc*, encoding the PSII reaction center protein, was significantly up-regulated. The qRT-PCR results revealed a similar trend in gene expression for *nppsbl* and *nppsbc*. The high upregulation of photosynthetic proteins reflects the overall surge in photosynthetic activity of their cells. When the saline-alkali concentration does not exceed the tolerance level of *Nitzschia palea*, this may increase the effective supply of CO_2_ for photosynthesis. This may be due to the large amount of (HCO_3_^−^ and CO_3_^2−^) inorganic carbon in the culture medium as the main source of substrate, accelerating the growth of algae cells. However, when the saline-alkali concentration exceeds the tolerance level of *Nitzschia palea*, damage or partial degradation of the light-trapping pigment protein comple [48].

### Effects of saline-alkali stress on the SOS pathway in *Nitzschia palea*

Saline-alkali stress induces the SOS pathway in microalgae cells. Calcium ion (Ca^2+^) mainly stimulates the SOS pathway, which is also essential for the regulation of Na^+^/K^+^ homeostasis in plants [49]. The SOS pathway is responsible for Na^+^ efflux in the cell and mainly involves three proteins, SOS1, SOS2 and SOS3, as Ca^2+^ sensors; SOS1 is a Na^+^/H^+^ retrograde transporter protein located in the cell membrane, and the expression of genes related to SOS1 is regulated by SOS3 and SOS2 [50]. The distribution of SOS2 proteins in the cytoplasmic membrane is beneficial for improving plant resistance to salt stress, and that SOS2 proteins are critical for coordinating the response of *Arabidopsis* roots and shoots to salt stress [51, 52]. Leaf and root tissues of rice induced up-regulated expression of the SOS2 gene under salt stress, and the expression abundance increased with increasing salinity levels [53, 54]. In this study, the results showed that the expression of *npsos1, npsos2* and * npsos3* proteins were significantly up-regulated under saline-alkali treatment; the qRT-PCR results showed the same trend in their gene expression. It has been observed that when the concentration of saline-alkali stress does not exceed the tolerance level of *Nitzschia palea*, the receptors on the algal cell membrane uptake extracellular Na^+^. Stimulating the regulation of SOS1, SOS2 and SOS3 proteins. Concurrently, the stimulation of Ca^2+^ signals prompt the exclusion of extracellular Na^+^ from SOS3 and SOS2, leading to exocytosis and maintaining ionic homeostasis within the cell. When the concentration of saline-alkali stress exceeds the tolerance level of *Nitzschia palea*, the receptor proteins and ion channels in the cell membrane may be disrupted, resulting in the inhibition of regulatory proteins involved in Ca^2+^ signaling and the inability to stimulate Ca^2+^ production. This phenomenon was also observed in proteomic analyses, wherein the proteins *nppmca* and *npant* in the calcium signaling pathway were significantly down-regulated.

### Effects of saline-alkali stress on osmoregulatory substances and antioxidant enzyme activities in *Nitzschia palea*

Saline and alkaline stress alter the intracellular osmotic pressure of microalgae, causing damage to the biofilm [55, 56]. When intracellular osmotic balance is disrupted, CO_2_ fixation is affected, enzyme activity is reduced and protein biosynthesis is interfered with, thereby inhibiting the growth of microalgae. The content of MDA can indicate the occurrence of lipid peroxidation in the cell, indirectly reflecting the extent of cell membrane damage. It was shown that *M. aeruginosa* showed a significant increase in MDA content under the combined stress of salinity and nanoparticulate matter, which led to lipid peroxidation in the cell membrane [57]. In this study, the MDA contents of *Nitzschia palea* increased after 4 days of saline-alkali stress, indicating that lipid peroxidation occurs in the cell membranes of the microalgae. The soluble sugar content decreased, the soluble protein content decreased, and the proline content increase. This indicates that the integrity of the cell membrane of *Nitzschia palea* is disrupted, leading to a decrease in intracellular osmotic regulation ability and thus accelerating the death of microalgae cells. Proteomic analyses revealed a significant up-regulation of *npcdk1*, a protein in the cellular senescence pathway, as well as a significant down-regulation of *npant*, a protein involved in ROS development. Studies have shown that microalgae produce large amounts of ROS under saline-alkali stress, causing excessive ROS damage to the cellular structure, while antioxidant enzyme activity, such as SOD, tends to decline [58–61]. However, microalgae scavenge excess ROS by inducing their own enzymes [62]. In this study, SOD activity decreased, CAT and POD activity showed a trend of increasing at low concentrations and decreasing at high concentrations, H_2_O_2_ and superoxide anion content gradually increased, and GSH and AsA content gradually decreased. The response of antioxidant enzymes exhibited a similar pattern of change, which may be attributed to the large amount of ROS accumulated in the cells when *Nitzschia palea* was subjected to saline-alkali stress, and then antioxidant enzymes responded synergistically to eliminate ROS and inhibit the accumulation of H_2_O_2_ in the algal cells, so as to alleviate the oxidative damage suffered by the cells [63, 64]. Proteomic analysis revealed a significant down-regulation of *npgme*, a protein in the ascorbate pathway. It is possible that the decrease in cell wall biosynthesis-related proteins and the reduction in ascorbic acid accumulation contribute to the decreased salinity tolerance of *Nitzschia palea*. In the glutathione metabolic pathway, the expression of *nppgd*, a protein involved, was significantly upregulated. It is possible that GSH deficiency disrupts cellular redox homeostasis, leading to ROS accumulation and triggering cellular damage or even cell death.

### Effect of saline- alkali stress on the metabolic pathway in *Nitzschia palea*

Microalgae regulate many proteins to maintain basic metabolism in the body during saline-alkali stress. It has been shown that under salt stress *Arthrospira*, a total of 141 differentially expressed proteins were identified, and it was also found that among them, both heat stress proteins for short-term adaptation to adversity and ABC transporter proteins controlling substance transport contributed to the study of improving resistance to high salt stress [65]. *Pyropia haitanensis* inhibits the Embden-Meyerhof-Parnas pathway(EMP) under high salt stress by a decrease in phosphofructokinase, but an increase in triose phosphate isomerase for both PPP purposes alleviates this inhibition and provides substrates for the TCA cycle, which energizes *Pyropia haitanensis* [66, 67]. In this study, the main biologically essential metabolisms were the pentose phosphate pathway(PPP), EMP pathway and the TCA cycle. In the pentose phosphate pathway, the expression of protein *nppgd* was significantly up-regulated, leading to an increase in ribose 5-phosphate accumulation and promoting glyceraldehyde 3-phosphate accumulation. In the glycolytic pathway, the expression of proteins *npgapdh, nppgam* and *npeno* was significantly down-regulated, leading to a decrease in the accumulation of pyruvate, which in turn led to less synthesis of acetyl coenzyme A(acetyl-CoA). In the fat synthesis pathway, the expression of proteins *npfabg, npfabi*, etc. was significantly down-regulated, leading to a reduction in partial fatty acid synthesis; qRT-PCR results showed that the expression of *npfabg* gene showed the same trend. In the TCA cycle, the expression of proteins *npsdha, npsdhb*, etc. was significantly down-regulated, and the expression of proteins *npsucd, nplsc1*, etc. was significantly up-regulated, which would promote the accumulation of malonyl-coenzyme A, and could promote the fatty acid biosynthesis pathway. This is consistent with findings by other scholars that an increase in saline-alkali concentration promotes the accumulation of energy storage substances in microalgae, the synthesis of lipids. The accumulation of lipids paves the way for better adaptation of the microalgae itself to the saline environment. In this study, we also detected the accumulation of nine fatty acids by GC/MS, which also further confirmed the results of the proteomic analysis.

With the increasing salinisation of water bodies and scarcity of freshwater resources, algae such as *Microcystis aeruginosa*, *Scenedesmus obliquus*, *Isochrysis galbana Parke* and *Tribonema minus* are considered as the best raw materials for the development of sustainable energy production [68, 69]. Studies have shown that *Microcystis aeruginosa* has a simple composition of lipids and fatty acids that can be easily transesterified to biodiesel [70]. Palmitic acid and oleic acid as the main components of *Scenedesmus obliquus* make it a suitable feedstock for biodiesel production [71]. The content of palmitoleic acid in the oil of *Tribonema minus* accounts for about 50% of the total fatty acids, which is much higher than that of other common microalgae [72]. In our study, we observed a significant accumulation of palmitic acid, palmitoleic acid, and oleic acid in *Nitzschia palea* as the saline-alkali concentration increased, suggesting its potential as an energy-rich algal strain. Due to the difficulty of purifying freshwater diatoms for culture and expanding them industrially, the oil production of diatoms is low compared to Chrysophyta and Chlorophyta. In contrast, *Nitzschia palea* has the advantage of rapid reproduction and is rich in oil compared to other species of diatoms, and the components that contribute to the accumulation of oil are slightly different(Additional file 1: Figure S6) Meanwhile, freshwater oil-producing diatoms applied to microalgae biodiesel include *Nitzschia, Ulnaria*, and *Navicula* [73, 74]. However, the cost of the production process is often high, whereas the microalgae culture in this study is conducive to reducing the cost of the preparation process, thus making it more convenient to be one of the candidate algal strains for bioenergy. Genetic and metabolic engineering to enhance lipid content in microalgae is now considered a prerequisite for economically competitive microalgal biofuel production [75]. Studies have shown that knockdown and partial silencing of *Nannochloropsis gadatana* by CRISPR/Cas9 technology facilitates the synthesis of lipids [76]. Overexpression of acetyl coenzyme A carboxylase (ACCase) in the chloroplasts of *Phaeodactylum tricornutum* has been shown to induce lipid overproduction [77]. The complete whole-genome sequencing of *Nitzschia palea* has been reported, providing a basis for future gene editing experiments and establishing it as a potential model species for freshwater diatoms. Cultivating *Nitzschia palea* under various saline concentrations not only increases biomass but also elevates oil content. In the future, the salinised water can be used to culture directly, which can greatly reduce the production cost and provide basic information for the aspect of freshwater diatom oil production as well as applying it to the production of microalgae biodiesel.

## Conclusion

In this study, the freshwater diatom *Nitzschia palea* has an innate saline-alkali tolerance mechanism to adapt different saline-alkali concentration. When the saline concentration remained below 90 mM, it promoted the growth of *Nitzschia palea* and the content of microalgal lipids and the synthesis of fatty acid substances, such as palmitic, palmitoleic and oleic acids. However, when the concentration of saline exceeded 90 mM, the destruction of antioxidant enzymes and membrane lipid peroxidation of microalgae cells intensified, resulting in the accumulation of ROS and the death of microalgae cells. According to the proteomics analysis, it involves major participation in the photosynthesis and carbon fixation pathway, glycolysis pathway, TCA cycle and fatty acid synthesis pathways. This study reveals for the proteome of the freshwater diatom *Nitzschia palea* and provides the basis for research data to become a model algal species for freshwater diatoms.

### Supplementary Information

**Additional file 1: Table S1.** Effects of different saline-alkali concentrations on the fatty acid composition of *Nitzschia palea*. **Table S2.** Protein sample concentration detection. **Figure S1.** GC–MS analysis of *Nitzschia palea* under saline-alkali stress. **Figure S2.** SDS-PAGE detection of *Nitzschia palea* protein. M: marker (250KD); 1–3: 90 mM; 4–6:60 mM; 7–9:30 mM; 10–12:CK. **Figure S3.** Basic information of the proteomes of *Nitzschia palea*. (A) peptide length distribution; (B) peptide coverage; (C) protein molecular mass distribution; (D) database comparison basic information. **Figure S4.** Differential expression protein heat map of Rhabdomonas oryzae treated with different saline-alkali concentrations. **Figure S5.** Subcellular localization prediction of differential proteins. **Figure S6.** Oil content of different types of microalgae(B and A, 2006; Ban Jianjiao, 2015; Chiu et al., 2009; Demirbas, 2009; Feinberg, 1984; Gouveia and Oliveira, 2008; Hongying X, 2023; Morais and Costa, 2007; Peng et al., 2001; Rodolfi et al., 2010; Sancho et al., 1999; Spolaore et al., 2006; Ting S, 2017; Ugwu et al., 2008; XiaMin J 2003; Yingmei Chen, 2021).

## Data Availability

All data generated or analyzed during this study are included in this published article.
