# Supplementary material for: Differential protein analysis of saline-alkali promoting the oil accumulation in Nitzschia palea
Source: Biotechnol Biofuels Bioprod. 2024 Jan 28;17:11. doi: 10.1186/s13068-023-02451-8 (PMC10823674; doi:10.1186/s13068-023-02451-8)
Supplement: Supplementary file 1 — Additional file 1: Table S1. Effects of different saline-alkali concentrations on the fatty acid composition of Nitzschia palea. Table S2. Protein sample concentration detection. Figure S1. GC–MS analysis of Nitzschia palea under saline-alkali stress. Figure S2. SDS-PAGE detection of Nitzschia palea protein. M: marker (250KD); 1–3: 90 mM; 4–6:60 mM; 7–9:30 mM; 10–12:CK. Figure S3. Basic information of the proteomes of Nitzschia palea. (A) peptide length distribution; (B) peptide coverage; (C) protein molecular mass distribution; (D) database comparison basic information. Figure S4. Differential expression protein heat map of Rhabdomonas oryzae treated with different saline-alkali concentrations. Figure S5. Subcellular localization prediction of differential proteins. Figure S6. Oil content of different types of microalgae(B and A, 2006; Ban Jianjiao, 2015; Chiu et al., 2009; Demirbas, 2009; Feinberg, 1984; Gouveia and Oliveira, 2008; Hongying X, 2023; Morais and Costa, 2007; Peng et al., 2001; Rodolfi et al., 2010; Sancho et al., 1999; Spolaore et al., 2006; Ting S, 2017; Ugwu et al., 2008; XiaMin J 2003; Yingmei Chen, 2021). [file 13068_2023_2451_MOESM1_ESM.docx]

Table S1 Components of culture medium WC

| Composition | Concentration(mg·L^-1^) |
| --- | --- |
| CaCl_2_·2H_2_O | 36.76 |
| MgSO_4_·7H_2_O | 36.97 |
| NaHCO_3_ | 12.60 |
| K_2_HPO_4_ | 8.71 |
| NaNO_3_ | 85.01 |
| Na_2_SiO_3_·9H_2_O | 28.42 |
| Na_2_EDTA | 4.36 |
| FeCl_3_·6H_2_O | 3.15 |
| CuSO_4_·5H_2_O | 0.01 |
| ZnSO_4_·7H_2_O | 0.022 |
| CoCl_2_·6H_2_O | 0.01 |
| MnCl_2_·4H_2_O | 0.18 |
| Na_2_MoO_4_·2H_2_O | 0.006 |
| H_3_BO_4_ | 1.0 |
| Thiamin HCl | 0.1 |
| Biotin | 0.0005 |
| B_12_ | 0.0005 |

Table S2 primers used for qRT-PCR

| Gene ID | Forward Primer (5′ to 3′) | Reverse Primer (5′ to 3′) |
| --- | --- | --- |
| *npactin* | CAGGTGCGACACGAAGTT | CCGTGTTTCCTTCGCTCA |
| *nppsbc* | GCGACGGCTTCTTCCACA | GCGGCGGAGTAGTTGACCTG |
| *npfabg* | GCGACGGCTTCTTCCACA | GCGGCGGAGTAGTTGACCTG |
| *nppsbl* | AAGGGAAAGTTCTGGGTG | GACGCCTTGACATTATCC |
| *npsos1* | GTGAAGCACGAACAGAAC | CATGACAGCATAGGGAGTG |
| *npsos2* | CATCAGTCGCCCGTTGCT | CATTGAAGAGGCGGAGGC |
| *npsos3* | CATCAGTCGCCCGTTGCT | CATTGAAGAGGCGGAGGC |

Table S3 Effects of different saline-alkali concentrations on the fatty acid composition of *Nitzschia palea*

| **Number** | **Fatty acid** | **Molecular formula** | **Retention period** | **Start time** | **End time** | **CAS number** |
| --- | --- | --- | --- | --- | --- | --- |
| **1** | Dodecanoic acid | C_13_H_26_O_2_ | 10.94 | 10.82 | 10.117 | 111-82-0 |
| **2** | Pentadecanoic acid | C_16_H_32_O_2_ | 13.479 | 13.440 | 13.544 | 7132-64-1 |
| **3** | 9-Hexadecenoic acid | C_17_H_32_O_2_ | 14.628 | 14.489 | 14.755 | 1120-25-8 |
| **4** | Hexadecanoic acid | C_17_H_34_O_2_ | 14.974 | 14.755 | 15.031 | 112-39-0 |
| **5** | Heptadecanoic acid | C_18_H_36_O_2_ | 16.577 | 16.531 | 16.647 | 1731-92-6 |
| **6** | 9-Octadecenoic acid | C_19_H_36_O_2_ | 18.469 | 18.227 | 18.665 | 1927-62-3 |
| **7** | Methyl stearate | C_19_H_38_O_2_ | 19.081 | 18.931 | 19.115 | 112-61-8 |
| **8** | Eicosanoic acid | C_20_H_40_O_2_ | 24.918 | 24.861 | 24.976 | 1120-28-1 |
| **9** | Docosanoic acid | C_22_H_44_O_2_ | 29.361 | 29.314 | 29.394 | 929-77-1 |

Table S4 Protein sample concentration detection

| Sample name | CK-1 | CK-2 | CK-3 | 30mM-1 | 30mM-2 | 30mM-3 | 60mM-1 | 60mM-2 | 60mM-3 | 90mM-1 | 90mM-2 | 90mM-3 |
| --- | --- | --- | --- | --- | --- | --- | --- | --- | --- | --- | --- | --- |
| Concentration  (μg/μl) | 7.248 | 5.414 | 6.290 | 8.417 | 7.983 | 5.292 | 6.468 | 6.994 | 7.666 | 6.003 | 6.287 | 6.013 |
| Volumetric  (μl) | 1000.0 | 1000.0 | 1000.0 | 1000.0 | 1000.0 | 1000.0 | 1000.0 | 1000.0 | 1000.0 | 1000.0 | 1000.0 | 1000.0 |
| Total amount  (μg) | 7248.0 | 5414.0 | 6290.0 | 8417.0 | 7983.0 | 5292.0 | 6468.0 | 6994.0 | 7666.0 | 6003.0 | 6287.0 | 6013.0 |

Table S5 Proteins in response to changes in salt and alkali concentration in *Nitzschia palea*

| Glycolysis / Gluconeogenesis | | | | | | |
| --- | --- | --- | --- | --- | --- | --- |
| Accession | Protein Name | Gene Name | EC | A VS B | A VS C | A VS D |
| B7FSI3 | Triosephosphate isomerase | TPI | EC1.2.1.12.1 | 0.70 | 0.72 | 0.49 |
| Q9M7R3 | Glyceraldehyde-3-phosphate dehydrogenase | GapC2 | EC1.2.1.12.2 | 0.79 | 0.83 | 0.58 |
| B7FSI4 | Glyceraldehyde-3-phosphate dehydrogenase | GapC4 | EC1.2.1.12.3 | 0.76 | 0.87 | 0.66 |
| B7GEI2 | Phosphoglycerate mutase | PGAM_4 | EC5.4.2.11 | 0.82 | 0.78 | 0.67 |
| B7GEF2 | phosphopyruvate hydratase | Enolase | EC4.2.1.11 | 0.74 | 0.71 | 0.70 |
| B7FZG0 | Pyruvate kinase | PK4a | EC2.7.1.40 | 0.81 | 0.80 | 0.81 |
| B7FRD2 | fructose-bisphosphatase | FBPC2 | EC3.1.3.11 | 0.71 | 0.89 | 0.92 |
| Carbon fixation in photosynthetic organisms | | | | | | |
| A0A8J9TF24 | ribose-5-phosphate isomerase | PTTT1 | EC5.3.1.6 | 0.84 | 0.86 | 0.79 |
| F1SXA1 | phosphoenolpyruvate carboxylase | ptpepc1 | EC4.1.1.31 | 0.84 | 0.79 | 0.70 |
| B7G9Y7 | Aspartate transaminase | AAT_1 | EC2.6.1.1 | 0.99 | 1.18 | 1.34 |
| Pentose and glucuronate interconversions | | | | | | |
| B7GEB0 | NADP-dependent oxidoreductase domain-containing protein | PHATRDRAFT | EC1.1.1.2 | 0.87 | 0.85 | 0.79 |
| Pyruvate metabolism | | | | | | |
| F1SXA1 | phosphoenolpyruvate carboxylase | ptpepc1 | EC4.1.1.31 | 0.84 | 0.79 | 0.70 |
| Pentose phosphate pathway | | | | | | |
| B5Y3S6 | Transaldolase | TAL | EC2.2.1.2 | 0.90 | 0.82 | 0.93 |
| Glutathione metabolism | | | | | | |
| B7FY00 | Ribonucleoside-diphosphate reductase | RDR | EC1.17.4.1 | 1.12 | 1.27 | 1.31 |
| B7FXB5 | 6-phosphogluconate dehydrogenase, decarboxylating | 6PGDH | EC1.1.1.44 | 1.48 | 1.22 | 1.51 |
| Citrate cycle (TCA cycle) | | | | | | |
| B5Y5N6 | Succinate dehydrogenase [ubiquinone] flavoprotein subunit, mitochondrial | SDH1 | EC1.3.5.1.1 | 0.62 | 0.55 | 0.54 |
| B7GA40 | Succinate dehydrogenase [ubiquinone] iron-sulfur subunit | SDH2 | EC1.3.5.1.2 | 0.56 | 0.47 | 0.46 |
| B7FXA2 | Succinate--CoA ligase [ADP-forming] subunit beta | SUCLA2 | EC6.2.1.4 | 1.05 | 1.06 | 1.36 |
| Glyoxylate and dicarboxylate metabolism | | | | | | |
| B7FX73 | methylmalonyl-CoA mutase | MCM | EC5.4.99.2 | 0.89 | 0.79 | 0.77 |
| B7GCL6 | propionyl-CoA carboxylase | PCC | EC6.4.1.3.1 | 1.15 | 0.94 | 0.61 |
| A0A8J9X1Y7 | Acetyl-CoA carboxylase domain-containing protein | PTTT1_LOCUS11168 | EC6.4.1.3.2 | 0.91 | 0.78 | 0.74 |
| B7G518 | Isocitrate lyase | ICL | EC4.1.3.1 | 0.54 | 0.70 | 0.66 |
| B7G6Q6 | glutamine synthase | GLNA | EC6.3.1.2 | 1.35 | 1.38 | 1.33 |
| B5Y594 | Serine hydroxymethyltransferase | SHMT | EC2.1.2.1 | 1.10 | 1.36 | 1.37 |
| Fatty acid biosynthesis | | | | | | |
| A0A8J9S8F9 | Enoyl-[acyl-carrier-protein] reductase | InhA | EC1.3.1.9 | 0.75 | 0.68 | 0.54 |
| B7G1R8 | 3-oxoacyl-[acyl-carrier protein | FABG | EC1.1.1.100 | 0.79 | 0.78 | 0.71 |
| E6Y9B3 | Stearoyl-ACP desaturase | SAD | EC1.14.192 | 0.59 | 0.61 | 0.57 |


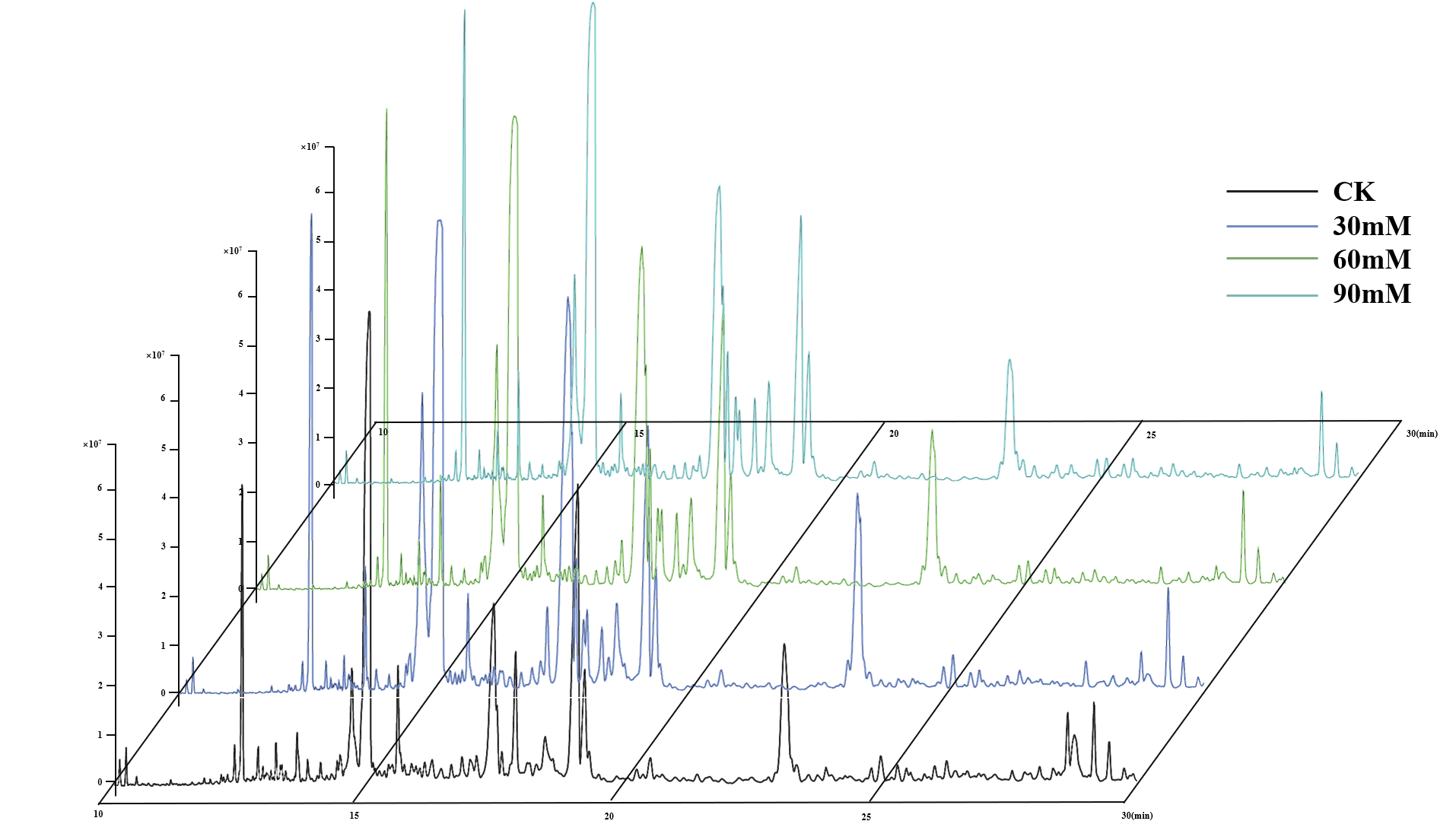


Figure S1 GC-MS analysis of *Nitzschia palea* under saline-alkali stress.


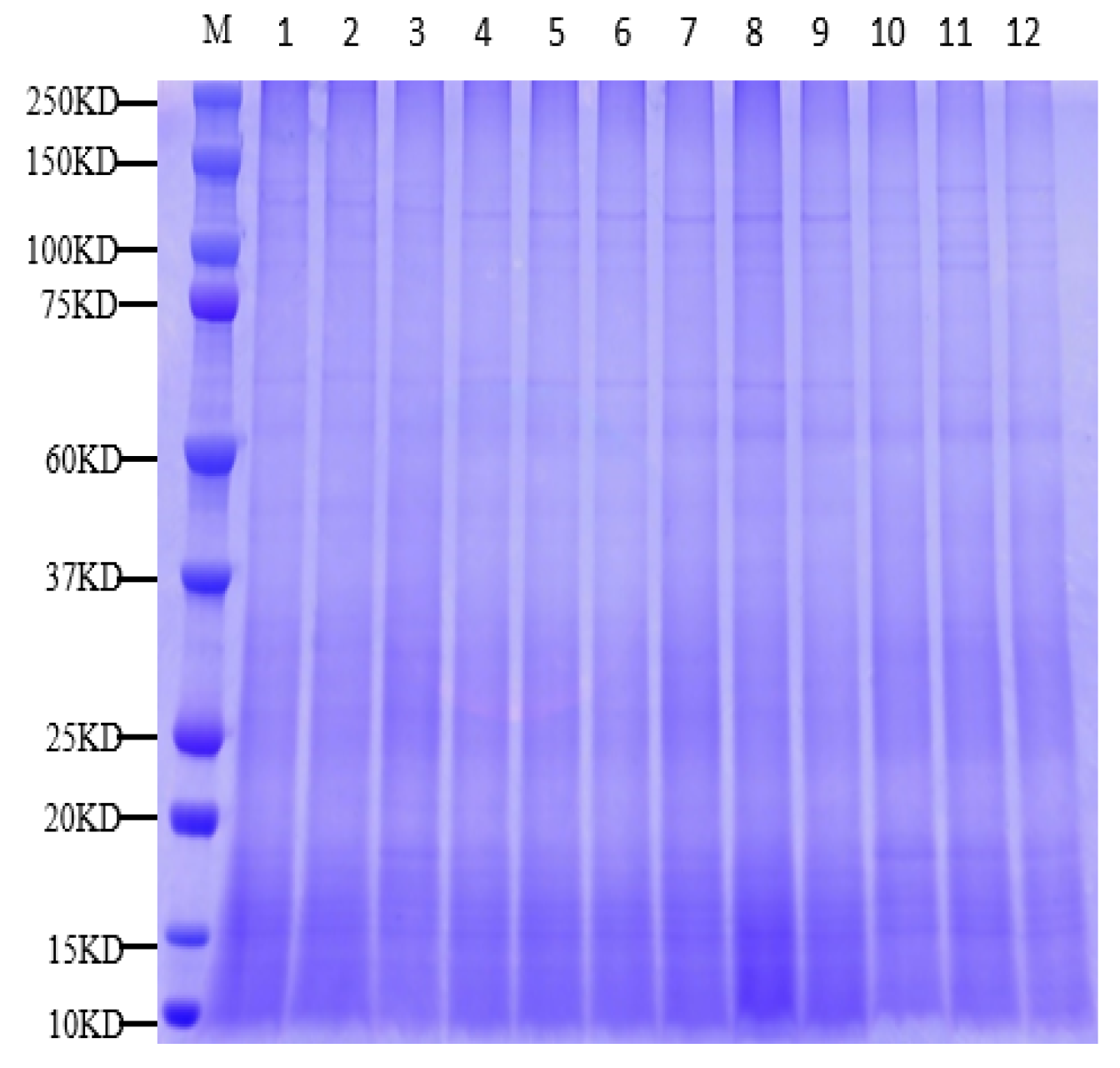


Figure S2 SDS-PAGE detection of *Nitzschia palea* protein. M：Marker (250KD)；1-3：90mM；4-6:60mM；7-9:30mM；10-12:CK


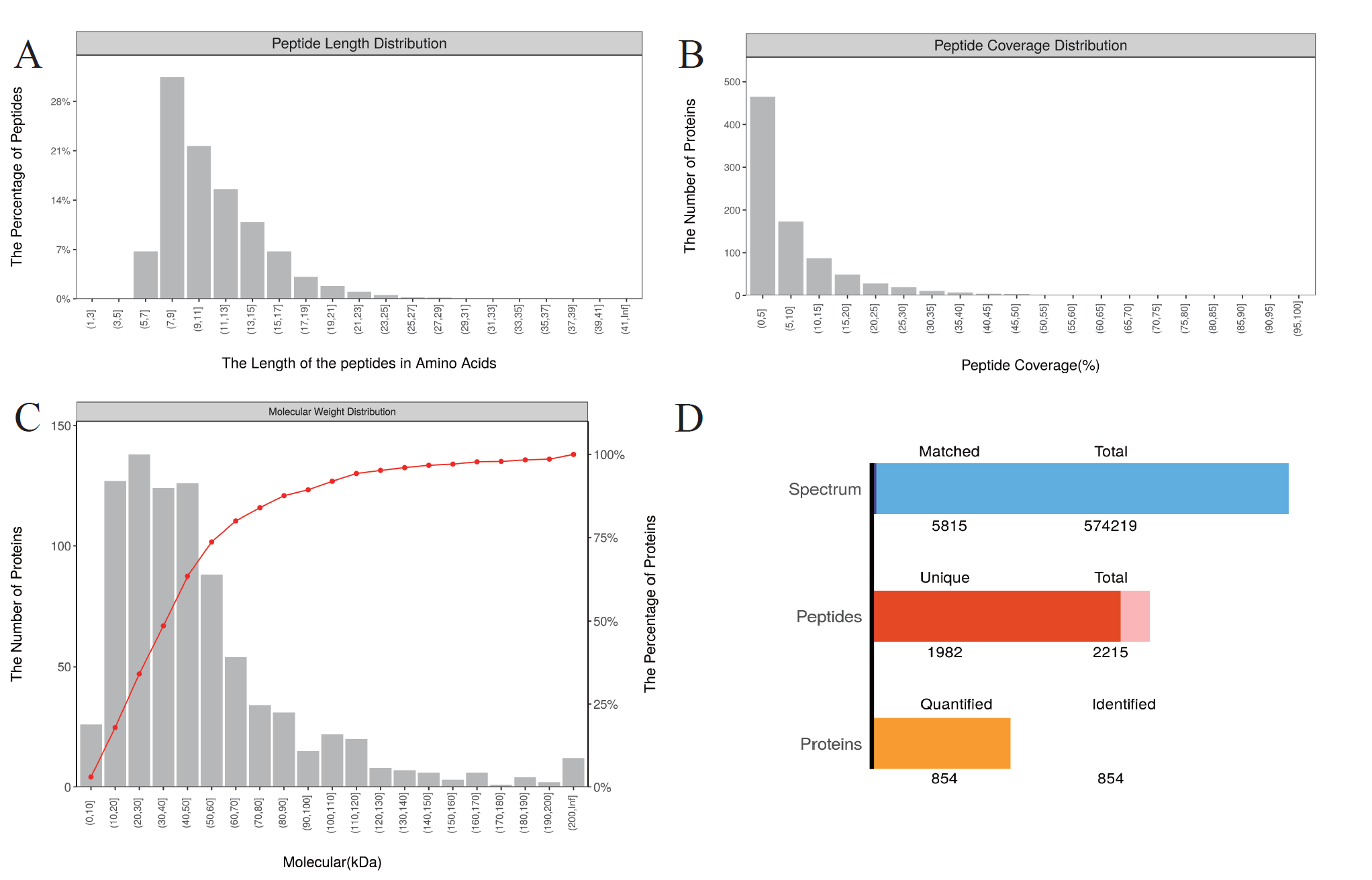


Figure S3 Basic information of the proteomes of *Nitzschia palea*. (A) peptide length distribution; (B) peptide coverage; (C) protein molecular mass distribution; (D) database comparison basic information


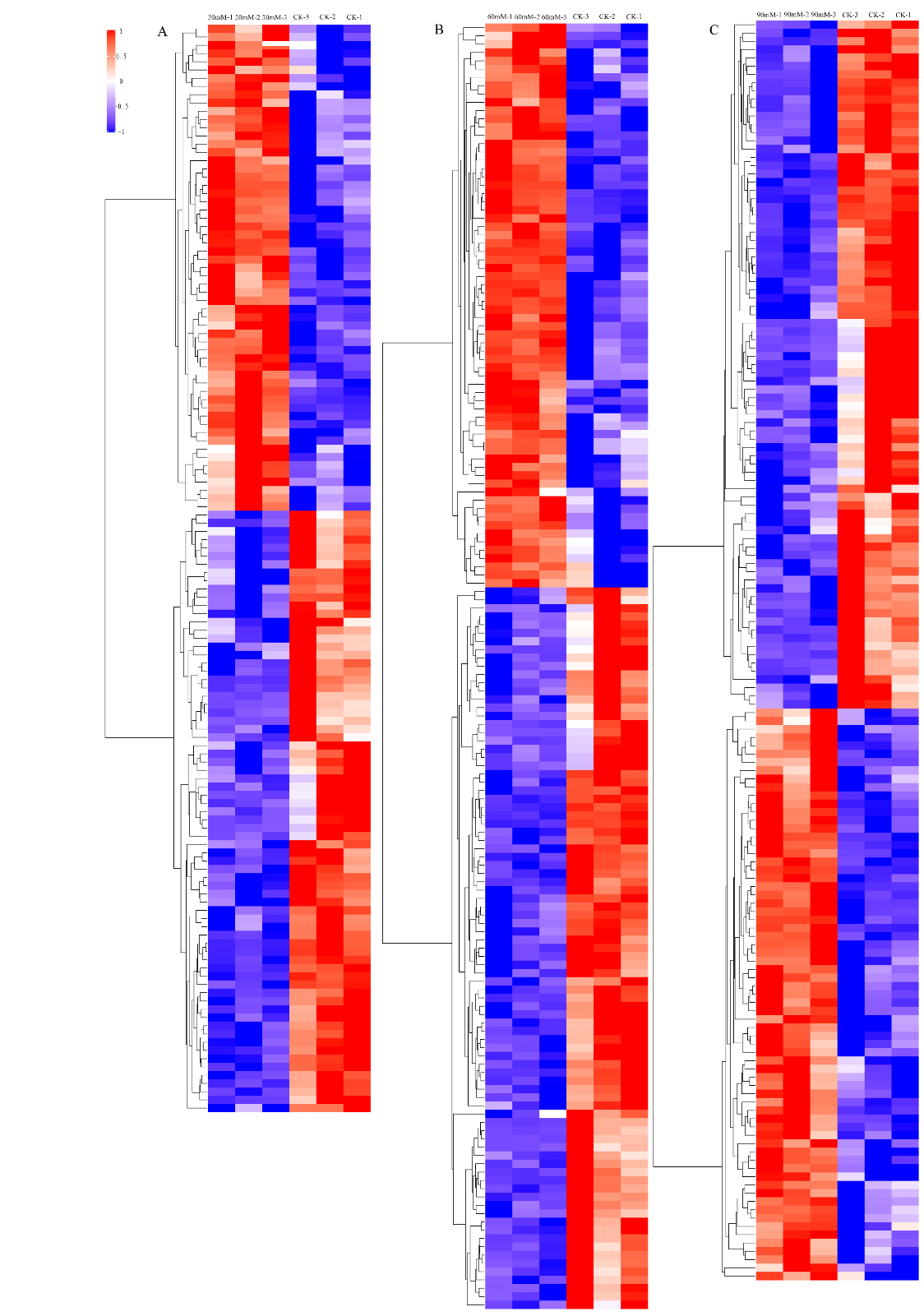


Figure S4 Differential expression protein heat map of *Nitzschia palea* treated with different saline-alkali concentrations


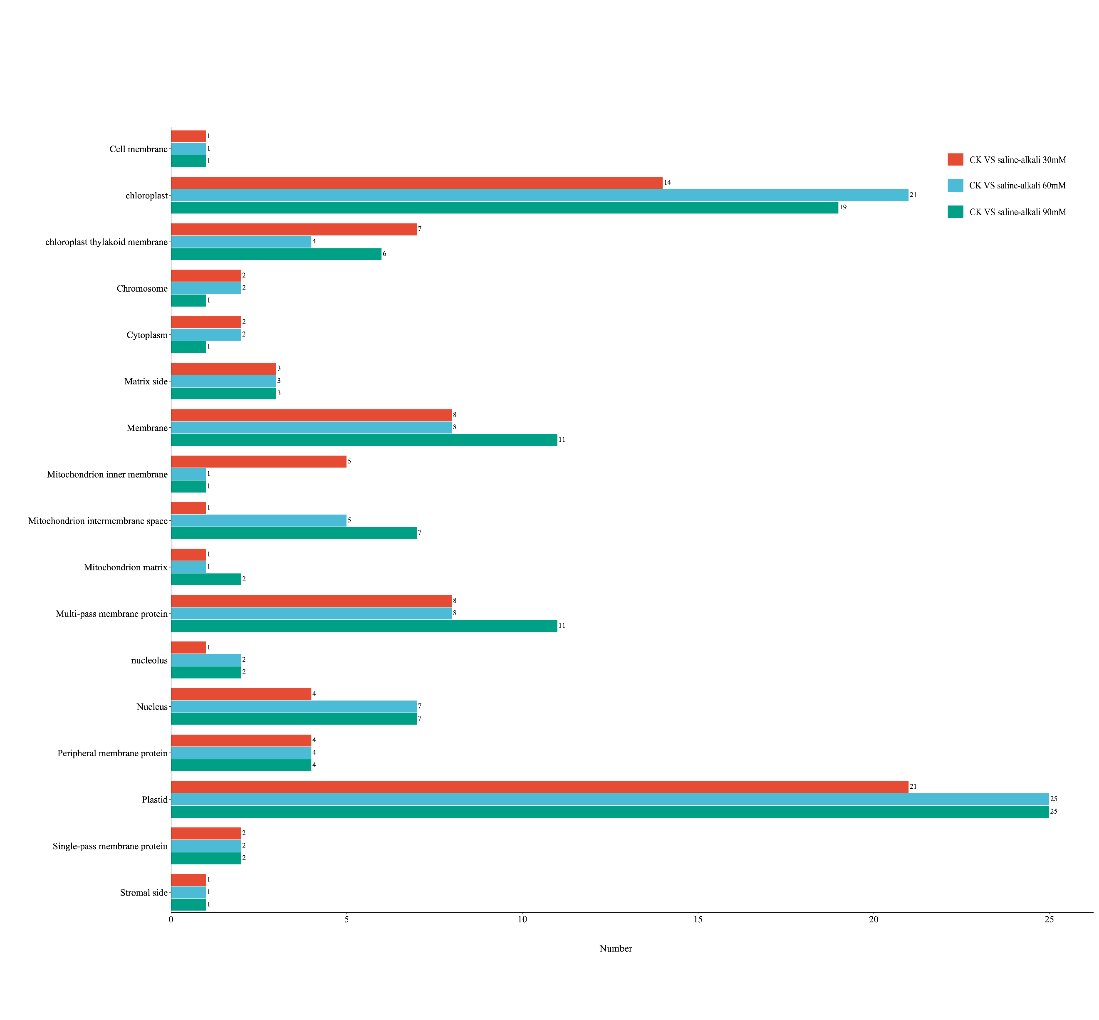


Figure S5 Subcellular localization prediction of differential proteins.


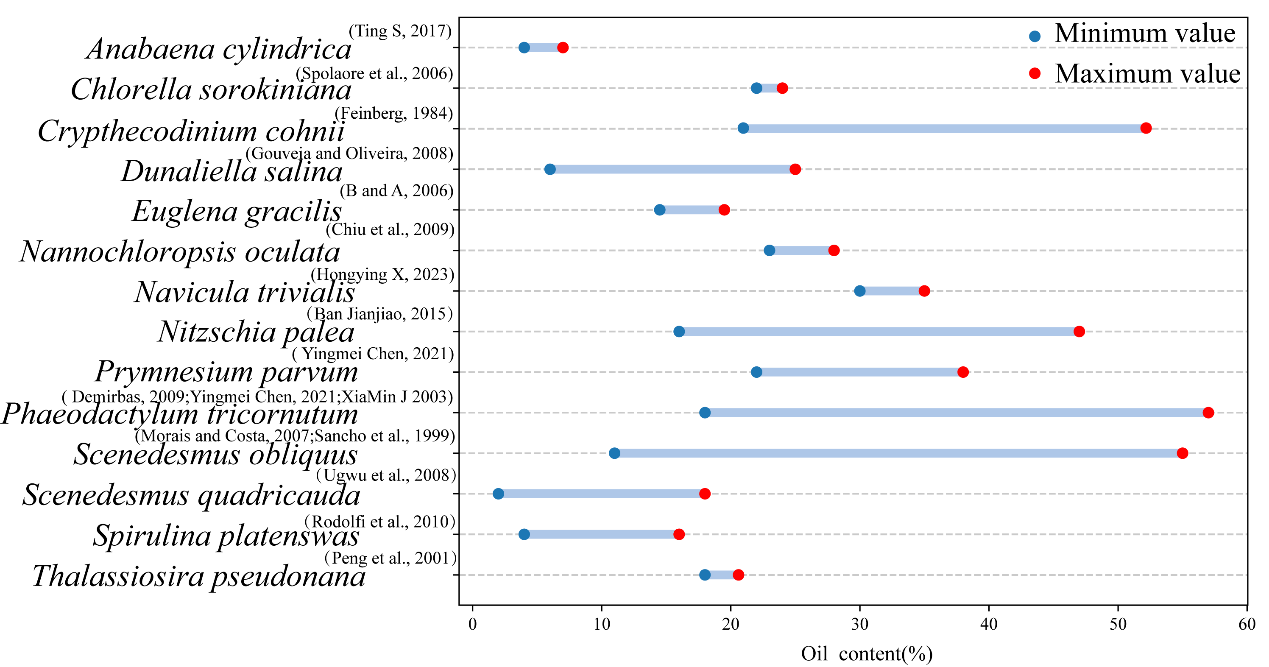


Figure S6 Oil content of different types of microalgae^(B and A, 2006; Ban Jianjiao, 2015; Chiu et al., 2009; Demirbas, 2009; Feinberg, 1984; Gouveia and Oliveira, 2008; Hongying X, 2023; Morais and Costa, 2007; Peng et al., 2001; Rodolfi et al., 2010; Sancho et al., 1999; Spolaore et al., 2006; Ting S, 2017; Ugwu et al., 2008; XiaMin J 2003; Yingmei Chen, 2021)^.

References

B, X.M.A., A, Q.W., 2006. Biodiesel production from heterotrophic microalgal oil. Bioresource Technology 97, 841-846.

Ban Jianjiao, F.J., Wang Zhiqiang, Xie Shulian, 2015. Effects of physical and chemical factors on the growth and neutral lipid contentof *Nitzschia palea*. ACTA ECOLOGICA SINICA 35, 8.

Chiu, S.Y., Kao, C.Y., Tsai, M.T., Ong, S.C., Chen, C.H., Lin, C.S., 2009. Lipid accumulation and CO2 utilization of Nannochloropsis oculata in response to CO2 aeration. Bioresource Technology 100, 833-838.

Demirbas, A., 2009. Progress and recent trends in biodiesel fuels. Energy Conversion & Management 50, 14-34.

Feinberg, D.A., 1984. Fuel options from microalgae with representative chemical compositions. Biomass Fuels, 30-35.

Gouveia, L., Oliveira, A.C., 2008. Microalgae as a Raw Material for Biofuels Production. Journal of Industrial Microbiology 36, 269-274.

Hongying X, J.L., XintongW, Yan L , Yawen F,Xinxin L , Fengyang S, 2023. SALINITY AND PH ON THE GROWTH AND TOTAL LIPID

CONTENT OF NAVICULA TRIVIALIS. ACTA HYDROBIOLOGICA SINICA, 1-10.

Morais, M., Costa, J., 2007. Carbon dioxide fixation by Chlorella kessleri, C-vulgaris, Scenedesmus obliquus and Spirulina sp cultivated in flasks and vertical tubular photobioreactors. Biotechnology Letters 29, 1349-1352.

Peng, W.M., Wu, Q.Y., Tu, P.G., Zhao, N.M., 2001. Pyrolytic characteristics of microalgae as renewable energy source determined by thermogravimetric analysis. Bioresource Technology 80, 1-7.

Rodolfi, L., Zittelli, G.C., Bassi, N., Padovani, G., Biondi, N., Bonini, G., Tredici, M.R., 2010. Microalgae for oil: strain selection, induction of lipid synthesis and outdoor mass cultivation in a low-cost photobioreactor. Biotechnology & Bioengineering 102, 100-112.

Sancho, M.M., Castillo, J.M.J., Yousfi, F.E., 1999. Photoautotrophic consumption of phosphorus by Scenedesmus obliquus in a continuous culture. Influence of light intensity. Process Biochemistry 34, 811-818.

Spolaore, P., Joannis-Cassan, C., Duran, E., Isambert, A., 2006. Commercial applications of microalgae. Elsevier, 2-5.

Ting S, L.H., Wei R, 2017. Progress in the application of microalgae in biomass development. Green Science and Technology, 4.

Ugwu, C.U., Aoyagi, H., Uchiyama, H., 2008. Photobioreactors for mass cultivation of algae. Bioresour Technol 99, 4021-4028.

XiaMin J , Y.Z., 2003. TOTAL LIPID AND FATTY ACID COMPOSITION OF14SPECIES OF MIRCOALGAE. ACTA HYDROBIOLOGICA SINICA 27, 243-247.

Yingmei Chen, Z.W., Huilan Luo, Yan Qin, 2021. Research Progress of Biofuel Production

by Microalgae. Chemical Engineering and Technology 11, 196-206.
